# Construction of a novel prognostic scoring model for HBV-ACLF liver failure based on dynamic data

**DOI:** 10.1038/s41598-024-63900-4

**Published:** 2024-07-02

**Authors:** Qun Cai, Hao Wang, Mingyan Zhu, Yixin Xiao, Tingting Zhuo

**Affiliations:** 1 Department of Infectious Diseases and Liver Diseases, Ningbo Medical Center Lihuili Hospital, Affiliated Lihuili Hospital of Ningbo University, 1111 Jiangnan Rd., Ningbo, 315100 China; 2 The First Affiliated Hospital, Zhejiang University School of Medicine, Hangzhou, China

**Keywords:** HBV, ACLF, Prognostic score, Liver cirrhosis, Hepatitis B

## Abstract

Early prognostic assessment of patients with hepatitis B virus-related acute-on-chronic liver failure (HBV-ACLF) is important for guiding clinical management and reducing mortality. The aim of this study was to dynamically monitor the clinical characteristics of HBV-ACLF patients, thereby allowing the construction of a novel prognostic scoring model to predict the outcome of HBV-ACLF patients. Clinical data was prospectively collected for 518 patients with HBV-ACLF and randomly divided into training and validation sets. We constructed day-1, day-2, and day-(1 + 3) prognostic score models based on dynamic time points. The prognostic risk score constructed for day-3 was found to have the best predictive ability. The factors included in this scoring system, referred to as DSM-ACLF-D3, were age, hepatic encephalopathy, alkaline phosphatase, total bilirubin, triglycerides, very low-density lipoprotein, blood glucose, neutrophil count, fibrin, and INR. ROC analysis revealed the area under the curve predicted by DSM-ACLF-D3 for 28-day and 90-day mortality (0.901 and 0.889, respectively) was significantly better than those of five other scoring systems: COSSH-ACLF IIs (0.882 and 0.836), COSSH-ACLFs (0.863 and 0.832), CLIF-C ACLF (0.838 and 0.766), MELD (0.782 and 0.762) and MELD-Na (0.756 and 0.731). Dynamic monitoring of the changes in clinical factors can therefore significantly improve the accuracy of scoring models. Evaluation of the probability density function and risk stratification by DSM-ACLF-D3 also resulted in the best predictive values for mortality. The novel DSM-ACLF-D3 prognostic scoring model based on dynamic data can improve early warning, prediction and clinical management of HBV-ACLF patients.

## Introduction

Acute-on-chronic liver failure (ACLF) is a major syndrome that adversely affects global health and has a high rate of short-term mortality^1,2^. The accurate diagnosis and prognosis of ACLF are therefore crucial for reducing the mortality rate. Currently, the model for end-stage liver disease (MELD) and the MELD-sodium (MELD-Na) scoring systems are widely used to predict outcome in end-stage liver disease, as well as for the allocation of organs in the liver transplantation setting^3–5^. In order to more accurately predict mortality in ACLF patients, the CLIF consortium developed and validated the CLIF-C ACLF score^6^. However, in view of the geographical and etiological differences, we recently proposed a definition for hepatitis B virus-related ACLF (HBV-ACLF) based on the Chinese Group for the Study of Severe Hepatitis B (COSSH). Although this prognostic evaluation system has been widely recognized^7^, the above scoring models are focused on the baseline indicators at admission. In reality, the development of ACLF is a dynamic process and the patient's condition may progress at any time. Therefore, dynamic monitoring of changes in the patient's clinical characteristics are likely to prove more accurate for evaluating the severity and prognosis of ACLF.

Although the definitions and diagnostic criteria for ACLF vary, it is generally believed that timely and dynamic assessment of the clinical course of ACLF patients is crucial for avoiding ineffective treatment and for allowing rational selection for liver transplant (LT). Merion et al. showed that dynamic monitoring for the MELD score allows more accurate prognostication of HBV-ACLF^8^. Several studies have also shown that assessment of the CLIF-C ACLF score on the third day after ACLF diagnosis has more accurate short-term prognostic value than the score obtained at initial diagnosis^9–11^. Hence, the use of dynamic scoring models to more accurately assess the prognosis of ACLF patients has attracted widespread attention in recent years. It is well known that the clinical course of liver failure exhibits a dynamic pattern, regardless of whether the final outcome is improvement or death. A more accurate assessment of prognosis should help to improve the management of ACLF patients. The aim of the current study was therefore to develop a novel dynamic prognostic scoring model that allows the outcome of HBV-ACLF patients to be predicted with greater accuracy.

## Methods

### Study design

This study initially included 2079 patients with HBV-ACLF who were hospitalized at the First Affiliated Hospital of Zhejiang University School of Medicine from March 2018 to December 2021. After excluding patients who did not meet the inclusion criteria (described below) and patients lost to follow-up, 518 patients with HBV-ACLF were included in the final analysis. These were randomly divided into a training set of 362 cases and a validation set of 156 cases. Clinical data for the first and the third days was collected from patients in the training set, thereby allowing the development of a dynamic prognostic scoring model. The accuracy of this novel prognostic scoring model was then verified using data from the validation set. Clinical and follow-up data was extracted from electronic data capture systems and case report forms. The research protocol was approved by the Clinical Research Ethics Committee of the First Affiliated Hospital, Zhejiang University School of Medicine, and all research subjects or their representatives signed the informed consent form. All methods were performed in accordance with the relevant guidelines and regulations.

### Patient selection

**Figure 1:**
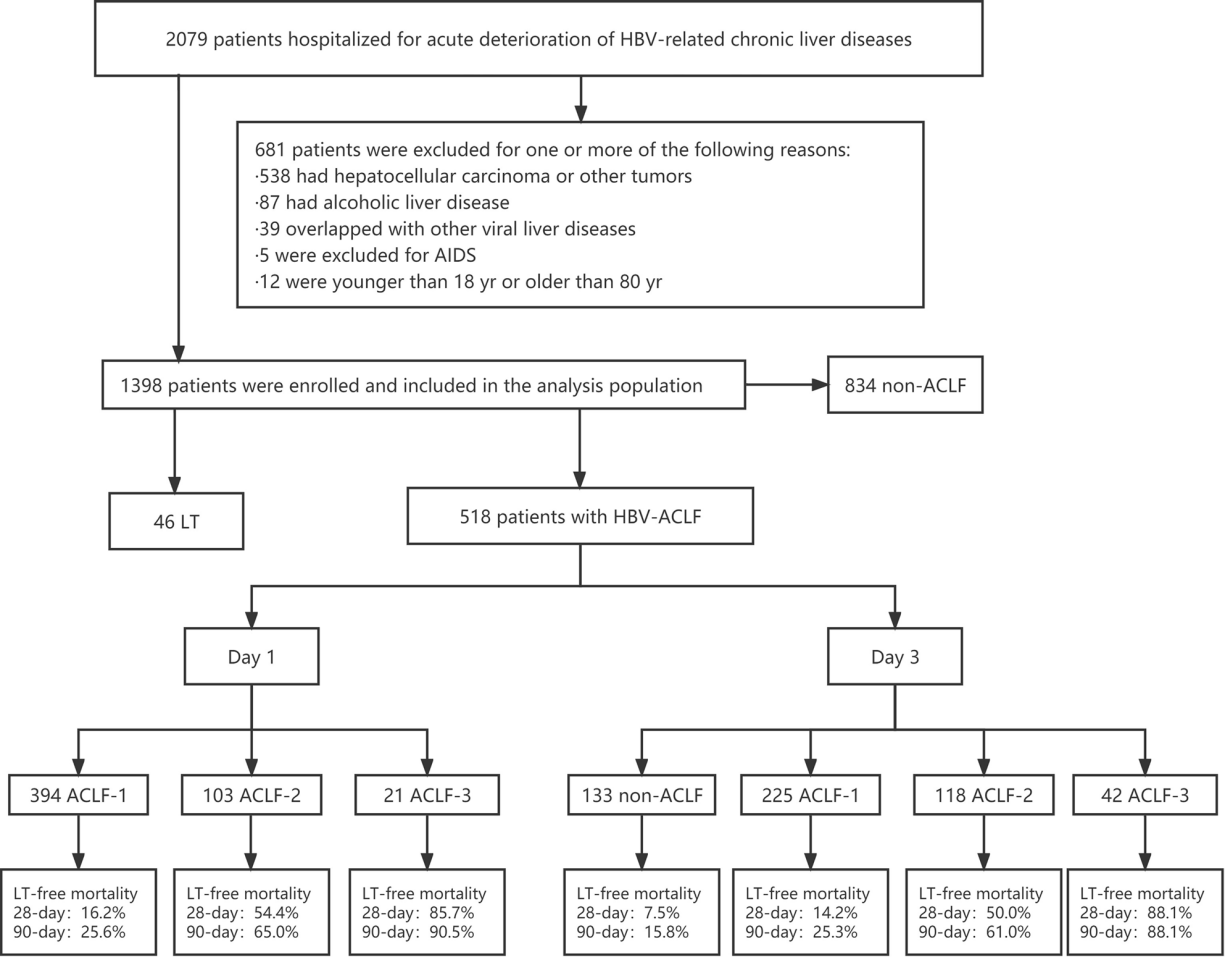
Screening, enrolment and classification of patients. ACLF, acute-on-chronic liver failure; HBV, hepatitis B virus; HBV-ACLF, HBV-related ACLF; LT, liver transplantation.

Patients who were hospitalized for at least 3 days with acute deterioration of HBV-related chronic liver disease were initially screened for this study. Acute deterioration was defined by the following COSSH criteria^7^: acute liver injury in patients with chronic hepatitis B (Total Bilirubin [TB] ≥ 5 mg/dl), or patients with hepatitis B cirrhosis and at least one of the symptoms of ascites, bacterial infection, hepatic encephalopathy (HE), upper gastrointestinal bleeding, or acute liver injury (TB ≥ 5 mg/dl). HE was defined and graded using the West Haven criteria. COSSH‐ACLF criteria was used to diagnose HBV‐ACLF, and this was divided into three grades (ACLF‐1, ACLF‐2, and ACLF‐3) as described previously^7^. The exclusion criteria are shown in Fig. 1. All enrolled patients received comprehensive medical treatment. Relevant clinical information and laboratory results were collected on the first and third days. Discharged patients were followed up at 28 days and 90 days after admission to confirm their survival and liver transplantation status.

### Scoring formulae

The COSSH-ACLF score was calculated using the formula: 0.741 × INR + 0.523 × HBV-sequential organ failure assessment score + 0.026 × age + 0.003 × TB (µmol/L)^12^. The COSSH-ACLF II score was calculated using the formula: 1.649 × INR + 0.457 × hepatic encephalopathy score + 0.425 × ln(neutrophil) + 0.396 × ln (TB (µmol/L)) + 0.576 × ln (serum urea) + 0.033 × age)^7^. The CLIF-C ACLF score was calculated using the formula: 10 × [0.33 × CLIF-organ failure score + 0.04 × age + 0.63 × ln (white blood cells) − 2]^13^. The MELD score was calculated using the formula: 3.78 × ln [TB (mg/dl)] + 11.2 × ln (INR) + 9.57 × ln [serum creatinine (mg/dl)] + 6.43 × etiology^14^. The MELD-Na score was calculated based on the MELD score using the formula: MELD score − Na − (0.025 × MELD score × (140 − Na)) + 140^15^.

### Development and validation of a dynamic prognostic score model for HBV-ACLF

Logistic regression was used to construct models for the variables on day-1, day-3, and day-(1 + 3). Before modeling, the data was randomly split into training and validation sets at a ratio of 7:3. LASSO regression was used to filter variables for the training set. Variables in which the LASSO regression coefficient was not zero were screened out and the remaining variables were used to develop a logistic regression model. The patient's risk score and short-term mortality were calculated according to the weight of the regression coefficient in the model. In both the training and validation sets, the area under the ROC curve (AUC) was used to evaluate the discrimination of the model. Calibration curves and decision curve analysis (DCA) were used to evaluate the calibration and clinical applicability of the model, respectively. Probability density curves of risk scores for the surviving and deceased groups were drawn, and overlapping portions of the curves for the two patient groups were compared. X-tile software was used to determine the optimal threshold value for the model risk score developed in this study. This was performed according to the largest log-rank test chi-square value, so as to achieve risk stratification (low risk, intermediate risk, and high risk).

### Statistical analysis

Data for the variables in this study are presented as the median (interquartile range, IQR), mean ± standard deviation (SD), or number of cases (percentage). Student's t-test or the Mann–Whitney U test was used to compare differences between two continuous variables, while the χ^2^ test was used to compare differences between categorical variables. SPSS software V.25 (SPSS, Chicago, Illinois, USA) was used to analyze and compare patient baseline characteristics. Other analyses were performed with R software version 4.0.3 (https://www.r-project.org), and a *p* value < 0.05 was considered statistically significant.

## Results

### Patient characteristics

The final study cohort comprised 518 patients with HBV-ACLF. Clinical data for the first day showed the following: 394 patients with ACLF-1 and liver transplant-free mortality rates (28-day and 90-day) of 16.2% and 25.6%, respectively; 103 patients with ACLF-2 and liver transplant-free mortality rates of 54.4% and 65.0%, respectively; 21 patients with ACLF-3 and liver transplant-free mortality rates of 85.7% and 90.5%, respectively. Following two days of comprehensive medical treatment for all patients included in the study, the clinical data was assessed again on the third day (day-3). The grades for the HBV-ACLF patients were found to have changed significantly, with 133 patients (25.7%) showing significant improvement and therefore included in the non-ACLF group. In addition, the 28-day and 90-day liver transplant-free mortality rates were 7.5% and 15.8%, respectively. The proportion of patients with ACLF-1 (n = 225) was lower compared to the first day (day-1), and the 28-day and 90-day liver transplant-free mortality rates were 14.2% and 25.3%, respectively. The proportion of patients with ACLF-2 (n = 118) was similar to the first day, and the 28-day and 90-day liver transplant-free mortality rates were 50.0% and 61.0%, respectively. The proportion of patients with ACLF-3 (n = 42) was significantly higher than on the first day, and the 28-day and 90-day liver transplant-free mortality rates were 88.1% and 88.1% respectively (Fig. 1).

### Baseline clinical features: day-1

The clinical data for patients on day-1 are shown in Table 1. Comparison of the ACLF-1, ACLF-2 and ACLF-3 patient groups revealed the following differences: a higher proportion of male patients (87.31%, 76.70%, and 80.90%, respectively); significantly different COSH-ACLF-II, COSSH-ACLF, CLIF-C ACLF, MELD and MELD-Na scores between the groups, indicating the ability of these scoring systems to evaluate the severity of disease; statistically significant differences in laboratory indicators including indirect bilirubin, glutamyl transpeptidase, serum urea, triglyceride, total cholesterol, high density lipoprotein, low density lipoprotein, very low density lipoprotein, white blood cells, neutrophils, monocytes, PT, INR, Fibrin, and D-dimer. Almost all patients in the three groups suffered liver failure (99.24%, 95.15% and 100.0%, respectively, for ACLF-1, ACLF-2 and ACLF-3). The incidence of coagulation failure was 0.25%, 78.64% and 100%, respectively. The incidence of brain failure was 0.25%, 18.45% and 76.19%, respectively. The incidence of renal failure was 0.25%, 5.83% and 23.81%, respectively, and the incidence of circulatory failure was 0%, 1.94% and 9.52%, respectively. Although the failure rate of various organs increased with ACLF grade, no significant difference in the rate of respiratory failure was observed according to ACLF grade.

**Table 1:** Clinical characteristics of the enrolled patients on day-1.

Characteristics	ACLF-1 (n = 394)	ACLF-2 (n = 103)	ACLF-3 (n = 21)	*P*
Male	344 (87.31%)	79 (76.70%)	17 (80.9%)	0.024
Age (years)	48.00 (39.00,57.00)	50.00 (42.00,59.00)	51.00 (43.00,53.00)	0.335
Vital signs				
MAP (mmHg)	84.00 (76.84,92.75)	85.67 (77.00,95.00)	82.63 (78.57,91.83)	0.626
SPO_2_ (%)	98.00 (97.00,99.00)	98.00 (96.00,99.00)	97.00 (96.50,98.00)	0.265
Complications				
Hepatic encephalopathy	1 (0.25%)	19 (18.45%)	16 (76.19%)	< 0.001
Hepatorenal syndrome	4 (1.02%)	9 (8.74%)	6 (28.57%)	< 0.001
I nfection	98 (24.87%)	28 (27。18%)	5 (23.81%)	0.880
Gastrointestinal haemorrhage	9 (2.28%)	4 (3.88%)	2 (9.52%)	0.125
Severity scores				
COSH-ACLF-IIs	5.62 ± 0.70	6.52 ± 0.67	7.16 ± 1.03	< 0.001
COSSH-ACLFs	5.75 (5.37,6.07)	6.50 (6.16,6.96)	7.32 (6.11,8.19)	< 0.001
CLIF-C ACLFs	38.27 ± 5.94	44.51 ± 6.00	45.29 ± 9.99	< 0.001
MELD	20.74 (19.02,22.68)	25.64 (23.41,28.44)	29.38 (23.59,36.21)	< 0.001
MELD-Na	21.83 ± 3.33	26.80 ± 4.97	31.09 ± 8.10	< 0.001
Laboratory data				
Total protein (g/L)	57.45 (53.30,62.00)	59.20 (54.40,64.10)	56.60 (53.95,63.55)	0.069
Globulin (g/L)	25.85 (22.08,30.40)	27.80 (23.30,32.90)	27.90 (24.45,33.85)	0.037
Alanine aminotransferase (U/L)	250.00 (103.75,543.50)	265.00 (99.00,718.00)	337.00 (107.50,657.00)	0.733
Aspartate aminotransferase (U/L)	158.50 (91.75,310.25)	190.00 (96.00,423.00)	234.00 (93.50,430.50)	0.313
Alkaline phosphatase (U/L)	138.00 (109.00,169.00)	147.00 (121.00,176.00)	145.00 (119.50,163.00)	0.165
Total bile acid (μmol/L)	250.25 (173.63,329.68)	251.00 (166.00,328.40)	247.60 (157.45,419.85)	0.888
Total bilirubin (μmol/L)	316.05 (251.80,398.93)	322.30 (258.00,428.00)	325.80 (273.85,445.20)	0.403
Direct bilirubin (μmol/L)	247.25 (198.45,317.23)	233.30 (184.40,308.70)	229.30 (184.10,308.45)	0.320
Indirect bilirubin (μmol/L)	62.95 (45.28,87.65)	84.30 (62.80,115.00)	108.10 (83.30,151.55)	< 0.001
Glutamyl transferase (U/L)	88.00 (62.00,129.50)	79.00 (49.00,117.00)	55.00 (35.00,68.50)	< 0.001
Creatinine (μmol/L)	62.00 (54.00,72.00)	65.00 (56.00,77.00)	79.00 (55.00,144.00)	0.012
Serum urea (mmol/L)	3.97 (2.96,5.22)	4.23 (2.90,6.28)	5.70 (2.89,14.68)	0.060
Triglyceride (mmol/L)	1.41 (1.10,1.85)	1.04 (0.91,1.26)	1.00 (0.90,1.10)	< 0.001
Total cholesterol (mmol/L)	2.27 (1.79,2.74)	1.90 (1.55,2.49)	1.62 (1.34,2.04)	< 0.001
High density lipoprotein (mmol/L)	0.18 (0.13,0.24)	0.23 (0.17,0.30)	0.24 (0.19,0.28)	< 0.001
Low density lipoprotein (mmol/L)	0.63 (0.32,1.13)	0.88 (0.55,1.35)	0.82 (0.35,1.18)	0.002
Very low density lipoprotein (mmol/L)	1.23 (0.85,1.73)	0.77 (0.42,1.00)	0.52 (0.37,0.93)	< 0.001
Glucose (mmol/L)	4.30 (3.58,5.47)	4.08 (2.87,5.21)	4.12 (3.14,6.20)	0.041
K (mmol/L)	4.05 (3.69,4.44)	4.04 (3.61,4.48)	4.09 (3.78,4.65)	0.645
Na (mmol/L)	138.00 (136.00,140.00)	138.00 (136.00,141.00)	138.00 (133.50,140.50)	0.571
Cl (mmol/L)	102.00 (100.00,105.00)	102.00 (99.00,105.00)	100.00 (95.50,105.00)	0.303
Ca (mmol/L)	2.07 (1.99,2.15)	2.07 (1.94,2.17)	2.06 (1.97,2.18)	0.918
P (mmol/L)	0.95 (0.81,1.10)	0.93 (0.78,1.06)	0.97 (0.76,1.32)	0.418
White blood cell (10^9^/L)	6.40 (4.70,8.60)	7.70 (5.80,9.50)	9.50 (6.95,12.80)	< 0.001
Neutrophil (10^9^/L)	4.30 (3.10,6.10)	5.20 (3.50,7.20)	7.20 (4.90,10.30)	< 0.001
Lymphocyte (10^9^/L)	1.10 (0.78,1.53)	1.16 (0.77,1.59)	1.24 (0.71,1.60)	0.861
Monocyte (10^9^/L)	0.66 (0.43,0.86)	0.79 (0.55,1.05)	1.01 (0.71,1.35)	< 0.001
Eosinophil (10^9^/L)	0.06 (0.03,0.10)	0.04 (0.01,0.10)	0.04 (0.01,0.10)	0.034
Basophil (10^9^/L)	0.02 (0.01,0.04)	0.02 (0.01,0.04)	0.02 (0.02,0.04)	0.243
Red blood cell (10^12^/L)	3.99 (3.52,4.43)	3.91 (3.35,4.36)	3.71 (3.09,4.51)	0.399
Haemoglobin (g/L)	128.00 (115.00,138.00)	122.00 (110.00,140.00)	117.00 (99.50,136.00)	0.104
Haematocrit (%)	36.40 (32.48,39.13)	34.90 (30.80,40.60)	32.90 (29.00,38.25)	0.062
Platelet count (10^9^/L)	102.00 (73.00,138.25)	105.00 (73.00,132.00)	131.00 (68.50,163.50)	0.489
INR	1.80 (1.59,2.03)	2.64 (2.45,3.00)	2.86 (2.66,3.34)	< 0.001
Fibrin (g/L)	1.44 (1.19,1.74)	1.02 (0.84,1.25)	0.86 (0.67,1.00)	< 0.001
Prothrombin time (s)	20.40 (18.20,22.80)	29.60 (26.40,32.60)	32.10 (29.20,36.90)	< 0.001
D-Dimer (ug/L)	1693.50 (808.75,3171.25)	3285.00 (1915.00,5293.00)	4158.00 (2824.00,5387.50)	< 0.001
Organ failures				
Liver	391 (99.24%)	98 (95.15%)	21 (100.00%)	0.009
Kidneys	1 (0.25%)	6 (5.83%)	5 (23.81%)	< 0.001
Coagulation	1 (0.25%)	81 (78.64%)	21 (100.00%)	< 0.001
Brain	1 (0.25%)	19 (18.45%)	16 (76.19%)	< 0.001
Circulation	0	2 (1.94%)	2 (9.52%)	< 0.001
Lungs	0	0	0	1.000
LT-free mortality				
28-day	64 (16.24%)	56 (54.37%)	18 (85.71%)	< 0.001
90-day	101 (25.63%)	67 (65.05%)	19 (90.48%)	< 0.001

Data are presented as the means ± SD, medians with (p25, p75), or numbers of patients (percentages).

ACLF, acute-on-chronic liver failure; MAP, Mean artery pressure; COSSH-ACLFs, COSSH-ACLF score; COSH-ACLF-IIs, COSSH-ACLF II score; CLIF-C ACLFs, CLIF Consortium ACLF score; MELD, Model for end-stage liver disease. LT, liver transplantation.

*P* value of comparisons across grades (ACLF-1, ACLF-2 and ACLF-3).

Table 2 shows differences in the day-1 clinical data between surviving and deceased HBV-ACLF patients. For 28-day mortality, the proportion of male patients was higher at 87.10% and 78.99%, respectively. The incidence of HE, hepatorenal syndrome, infection and GIH were significantly higher in the deceased group, as well as the scores for COSH-ACLF-II, COSSH-ACLF, CLIF-C ACLF, MELD and MELD-Na. In addition, the surviving and deceased patient groups had significantly different levels of AST, TB, indirect bilirubin, serum urea, triglyceride, total cholesterol, very low density, neutrophils, fibrin, INR, PT and D-Dimer. The clinical data for 90-day mortality showed the same trends as for 28-day mortality.

**Table 2:** Comparison of variable characteristics between the survival group and death groups on day-1.

Characteristics	28-day survival group (n = 380)	28-day death group (n = 138)	*P*	90-day survival group (n = 331)	90-day death group (n = 187)	*P*
Male	331 (87.10%)	109 (78.99%)	0.032	292 (88.22%)	148 (79.14%)	0.006
Age (years)	46.99 ± 11.54	54.38 ± 10.85	< 0.001	46.00 (38.00,55.00)	54.00 (47.00,60.00)	< 0.001
Therapies						
Vasopressors	0	4 (2.90%)	< 0.001	0	4 (2.14%)	0.008
Mechanical ventilation	0	0	1.000	0	0	1.000
Complications						
Hepatic encephalopathy	12 (3.16%)	24 (17.39%)	< 0.001	9 (2.72%)	27 (14.44%)	< 0.001
Hepatorenal syndrome	7 (1.84%)	12 (8.70%)	< 0.001	5 (1.51%)	14 (7.49%)	< 0.001
Infection	89 (23.42%)	42 (30.43%)	0.105	73 (22.05%)	58 (31.02%)	0.024
Gastrointestinal haemorrhage	5 (1.32%)	10 (7.25%)	< 0.001	5 (1.51%)	10 (5.35%)	0.012
Severity scores						
COSH-ACLF-IIs	5.61 ± 0.69	6.57 ± 0.78	< 0.001	5.54 ± 0.67	6.43 ± 0.80	< 0.001
COSSH-ACLFs	5.75 (5.37,6.07)	6.48 (6.07,6.94)	< 0.001	5.70 (5.33,6.03)	6.39 (5.90,6.84)	< 0.001
CLIF-C ACLFs	38.10 ± 5.86	44.47 ± 6.77	< 0.001	37.68 ± 5.77	43.54 ± 6.68	< 0.001
MELD	21.12 (19.23,23.49)	23.49 (20.51,27.47)	< 0.001	20.93 (19.12,23.00)	23.38 (20.44,26.90)	< 0.001
MELD-Na	22.09 (20.02,24.50)	24.73 (21.52,28.91)	< 0.001	21.85 (19.88,24.01)	24.25 (21.50,28.26)	< 0.001
Vital signs						
MAP (mmHg)	84.00 (76.67,92.95)	86.83 (79.48,96.08)	0.027	83.67 (76.67,92.00)	85.67 (78.00,95.00)	0.095
SPO2 (%)	98.00 (97.00,99.00)	98.00 (96.00,98.00)	0.003	98.00 (97.00,99.00)	98.00 (96.00,98.00)	0.024
Laboratory data						
Total protein (g/L)	57.75 (53.60,62.50)	58.25 (53.90,63.25)	0.599	57.70 (53.60,62.50)	58.10 (53.90,63.10)	0.834
Albumin (g/L)	31.20 (28.50,34.00)	30.40 (28.55,33.40)	0.306	31.40 (28.90,34.20)	30.30 (28.10,33.40)	0.027
Globulin (g/L)	26.15 (22.30,30.68)	26.55 (22.40,32.05)	0.323	25.80 (22.10,30.70)	27.20 (22.80,31.60)	0.168
Alanine aminotransferase (U/L)	250.00 (103.25,579.00)	279.50 (103.75,582.00)	0.634	262.00 (105.00,589.00)	267.00 (99.00,576.00)	0.802
Aspartate aminotransferase (U/L)	156.00 (91.00,284.75)	230.50 (96.00,430.00)	0.014	155.00 (92.00,283.00)	200.00 (95.00,420.00)	0.034
Alkaline phosphatase (U/L)	138.00 (109.00,167.75)	145.00 (116.00,176.00)	0.138	140.00 (109.00,168.00)	143.00 (114.00,175.00)	0.360
Total bile acid (μmol/L)	250.25 (174.13,325.18)	249.30 (164.83,347.63)	0.720	254.90 (176.30,330.50)	246.00 (164.30,336.80)	0.536
Total bilirubin (μmol/L)	306.90 (251.38,394.23)	345.05 (276.35,430.03)	0.004	297.40 (249.40,382.20)	348.90 (278.30,436.80)	< 0.001
Direct bilirubin (μmol/L)	239.10 (194.93,307.50)	263.45 (191.85,329.23)	0.158	232.80 (193.00,290.50)	267.00 (198.70,338.00)	0.003
Indirect bilirubin (μmol/L)	64.25 (46.60,88.40)	82.80 (57.10,115.25)	< 0.001	62.80 (45.40,87.50)	79.40 (56.80,113.30)	< 0.001
Glutamyl transferase (U/L)	87.00 (60.00,126.00)	76.00 (51.00,122.00)	0.072	87.00 (60.00,124.00)	76.00 (51.00,126.00)	0.161
Creatinine (μmol/L)	63.00 (56.00,73.75)	61.00 (51.00,77.00)	0.342	63.00 (56.00,73.00)	62.00 (53.00,77.00)	0.793
Serum urea (mmol/L)	3.80 (2.86,5.12)	4.72 (3.22,7.31)	< 0.001	3.74 (2.81,5.02)	4.67 (3.25,6.63)	< 0.001
Triglyceride (mmol/L)	1.36 (1.07,1.82)	1.09 (0.94,1.47)	< 0.001	1.37 (1.08,1.82)	1.14 (0.98,1.60)	< 0.001
Total cholesterol (mmol/L)	2.23 (1.76,2.71)	1.97 (1.56,2.64)	< 0.001	2.25 (1.80,2.70)	1.99 (1.55,2.68)	< 0.001
High density lipoprotein (mmol/L)	0.19 (0.13,0.24)	0.21 (0.15,0.28)	0.003	0.19 (0.13,0.24)	0.20 (0.15,0.28)	0.046
Low density lipoprotein (mmol/L)	0.70 (0.33,1.18)	0.74 (0.43,1.21)	0.312	0.70 (0.34,1.19)	0.69 (0.39,1.16)	0.771
Very low density lipoprotein (mmol/L)	1.18 (0.83,1.71)	0.81 (0.52,1.29)	< 0.001	1.20 (0.83,1.71)	0.90 (0.56,1.34)	< 0.001
Glucose (mmol/L)	4.16 (3.47,5.30)	4.51 (3.30,6.38)	0.157	4.19 (3.53,5.25)	4.37 (3.30,6.20)	0.304
K (mmol/L)	4.05 (3.69,4.44)	4.06 (3.58,4.50)	0.971	4.06 (3.71,4.44)	4.02(3.58,4.45)	0.477
Na (mmol/L)	138.00 (136.00,140.00)	138.00 (135.00,140.00)	0.140	138.00 (136.00,140.00)	138.00 (135.00,140.00)	0.155
Cl (mmol/L)	102.00 (100.00,105.00)	101.00 (98.00,104.00)	0.004	103.00 (100.00,105.00)	101.00 (99.00,104.00)	< 0.001
Ca (mmol/L)	2.07 (1.99,2.15)	2.06 (1.96,2.17)	0.415	2.07 (1.99,2.15)	2.06 (1.97,2.16)	0.429
P (mmol/L)	0.96 (0.83,1.11)	0.91 (0.74,1.09)	0.037	0.97 (0.83,1.11)	0.89(0.73,1.09)	0.001
White blood cell (10^9^/L)	6.50 (4.70,8.60)	7.45 (5.95,10.00)	0.001	6.50 (4.70,8.70)	7.20 (5.60,9.40)	0.008
Neutrophil (10^9^/L)	4.30 (3.00,6.20)	5.20 (3.70,7.25)	< 0.001	4.30 (3.00,6.10)	5.10 (3.50,7.00)	< 0.001
Lymphocyte (10^9^/L)	1.14 (0.82,1.59)	1.06 (0.69,1.45)	0.055	1.16 (0.84,1.61)	1.03 (0.70,1.43)	0.004
Monocyte (10^9^/L)	0.66 (0.44,0.88)	0.78 (0.54,1.05)	0.001	0.66 (0.44,0.88)	0.73 (0.51,1.02)	0.006
Eosinophil (10^9^/L)	0.06 (0.02,0.11)	0.05 (0.02,0.10)	0.056	0.06 (0.03,0.11)	0.05 (0.02,0.09)	0.059
Basophil (10^9^/L)	0.02 (0.01,0.04)	0.02 (0.01,0.04)	0.498	0.02 (0.01,0.04)	0.02 (0.01,0.04)	0.102
Red blood cell (10^12^/L)	4.00 (3.52,4.46)	3.84 (3.46,4.34)	0.073	4.00 (3.56,4.46)	3.91 (3.42,4.34)	0.081
Haemoglobin (g/L)	128.00 (113.00,138.00)	123.00 (112.00,137.00)	0.145	128.00 (115.00,138.00)	123.00 (110.00,137.00)	0.112
Haematocrit (%)	36.30 (32.40,39.20)	35.15 (31.10,39.00)	0.105	36.40 (32.80,39.20)	34.90 (31.10,39.20)	0.068
Platelet count (10^9^/L)	105.50 (73.00,139.75)	98.00 (65.00,132.50)	0.087	102.00 (74.00,143.00)	96.00 (65.00,130.00)	0.002
INR	1.82 (1.59,2.08)	2.38 (1.95,2.86)	< 0.001	1.80 (1.58,2.07)	2.21 (1.83,2.69)	< 0.001
Fibrin (g/L)	1.39 (1.13,1.71)	1.10 (0.90,1.57)	< 0.001	1.39 (1.13,1.76)	1.22 (0.93,1.57)	< 0.001
Prothrombin time (s)	20.80 (18.30,23.40)	26.70 (21.45,31.38)	< 0.001	20.50 (18.20,23.20)	25.10 (20.07,30.01)	< 0.001
D-Dimer (ug/L)	1615.50 (737.00,3099.25)	3255.00 (1967.50,5449.00)	< 0.001	1474.00 (711.00,2981.00)	3091.00 (1742.00,5101.00)	< 0.001

Data are presented as the means ± SD, medians with (p25, p75), or numbers of patients (percentages).

ACLF, acute-on-chronic liver failure; MAP, Mean artery pressure; COSSH-ACLFs, COSSH-ACLF score; COSH-ACLF-IIs, COSSH-ACLF II score; CLIF-C ACLFs, CLIF Consortium ACLF score; MELD, Model for end-stage liver disease. LT, liver transplantation.

### Development and validation of a novel prognostic score model based on day-1 clinical data

**Figure 2:**
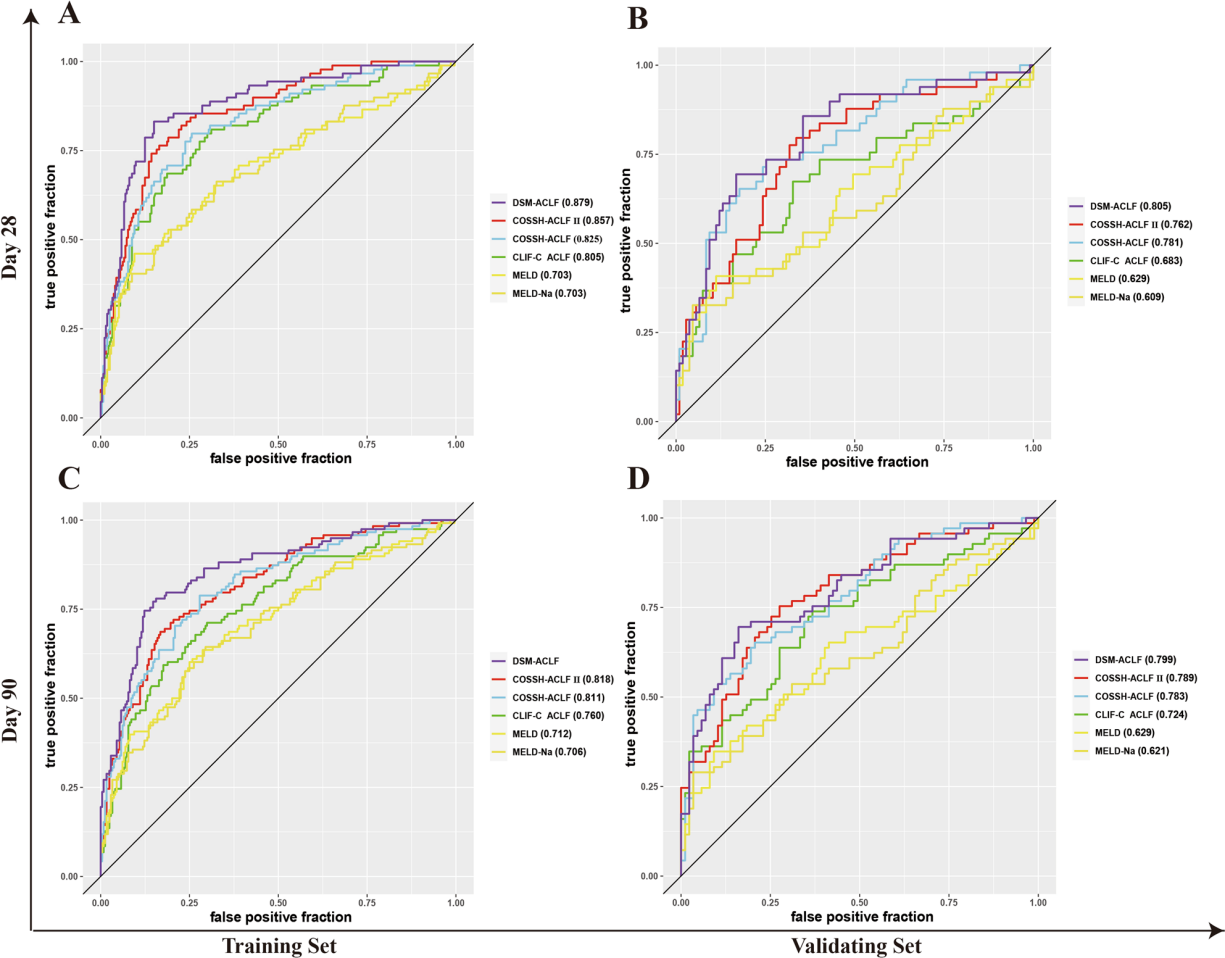
Time-dependent ROC curves of the new score and of the five other scores on day-1. (**A**, **B**) time-dependent ROC curves at 28 day since admission; (**A**, **C**) in the training set; (**C**,**D**) time-dependent ROC curves at 90 day since admission; (**B**,**D**) in the validation set.

With 28-day death as the outcome variable, the LASSO algorithm was used to screen 8 variables in the training set: age, HE, GIH, SPO2, serum urea, WBC, neutrophils and INR. Using a logistic regression model, a DSM-ACLF (day-1) score for HBV-ACLF patients was then constructed based on data from day-1 as follows: DSM-ACLF (day-1) = 7.910 + 0.074 × age + 1.679 × HE (with*1/without*0) + 1.600 × GIH (with *1/without *0) − 0.188 × SPO2 + 0.033 × serum urea + 0.132 × WBC + 0.017 × neutrophils + 1.871 × INR. Following ROC analysis, the AUCs at 28-day and 90-day follow-up for the DSM-ACLF (day-1) score and for the other five scoring systems evaluated in the training set were: DSM-ACLF (day-1), 0.879 and 0.857, respectively; COSSH-ACLF II, 0.857 and 0.818; COSSH-ACLF, 0.825 and 0.811; CLIF-C ACLF, 0.805 and 0.760; MELD, 0.703 and 0.712; MELD-Na, 0.703 and 0.706. These results indicate that all six models have good predictive value for the outcome of HBV-ACLF patients. However, the AUC for the DSM-ACLF (day-1) score was clearly higher than those of the other models, thus demonstrating better discrimination and predictive ability. This novel DSM-ACLF (day-1) scoring model was then applied to the validation set, where the AUCs for 28-day and 90-day ROC curves were found to be 0.805 and 0.799, respectively (Fig. 2). Calibration curves and decision curves for the six prognostic scoring systems are shown in Supplementary Fig. 1. The DSM-ACLF (day-1) score showed better calibration and net benefits than the other scoring systems in both the training and validation datasets.

**Figure 3:**
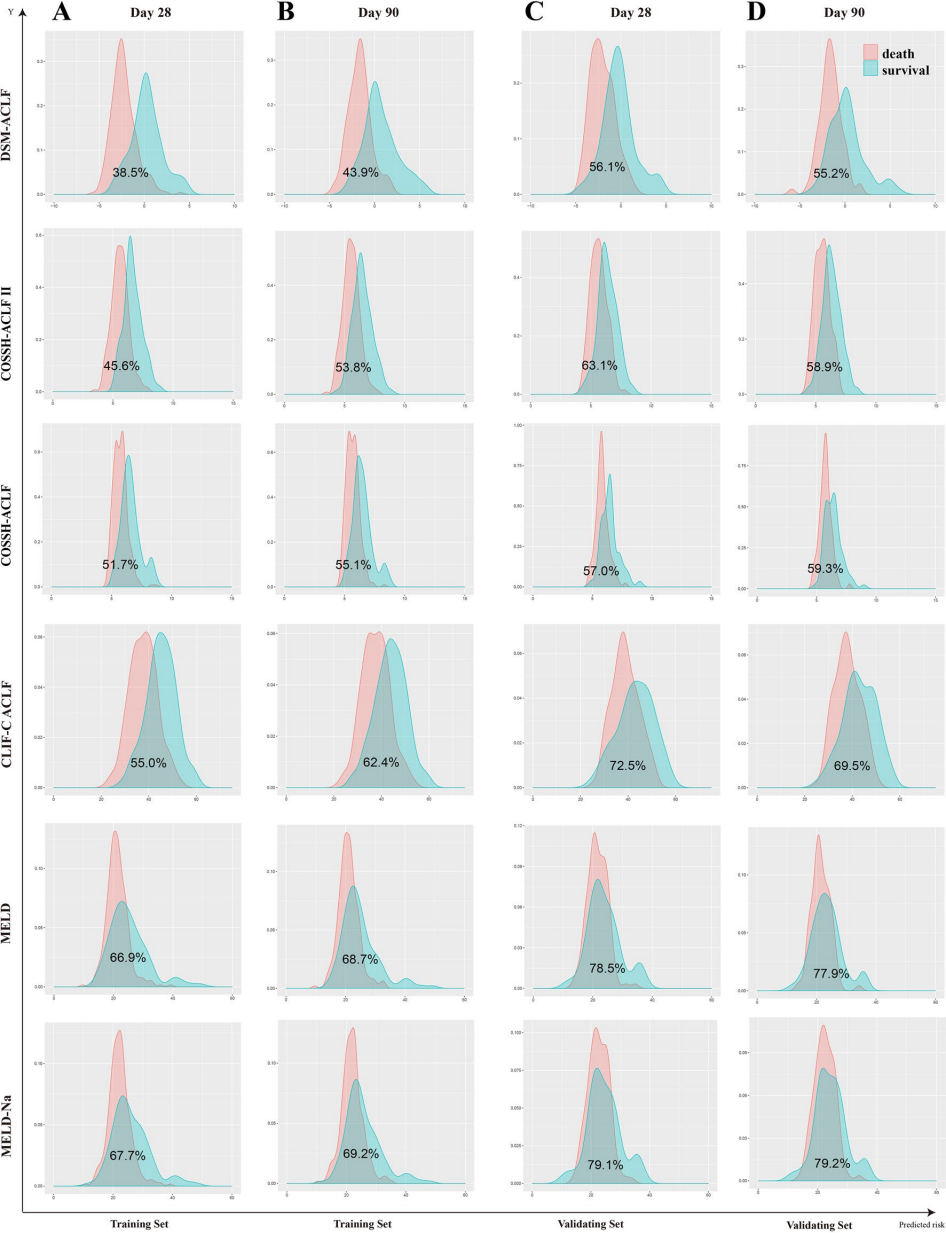
Probability density function (PDF) of the new score and of the five other scores on day-1. (**A**,**B**) in the training set; time-dependent ROC curves at 28 day since admission; (**A**,**C**) PDF at 28 day since admission; (**C**,**D**) in the validation set; (**B**,**D**) PDF at 90 day since admission.

**Figure 4:**
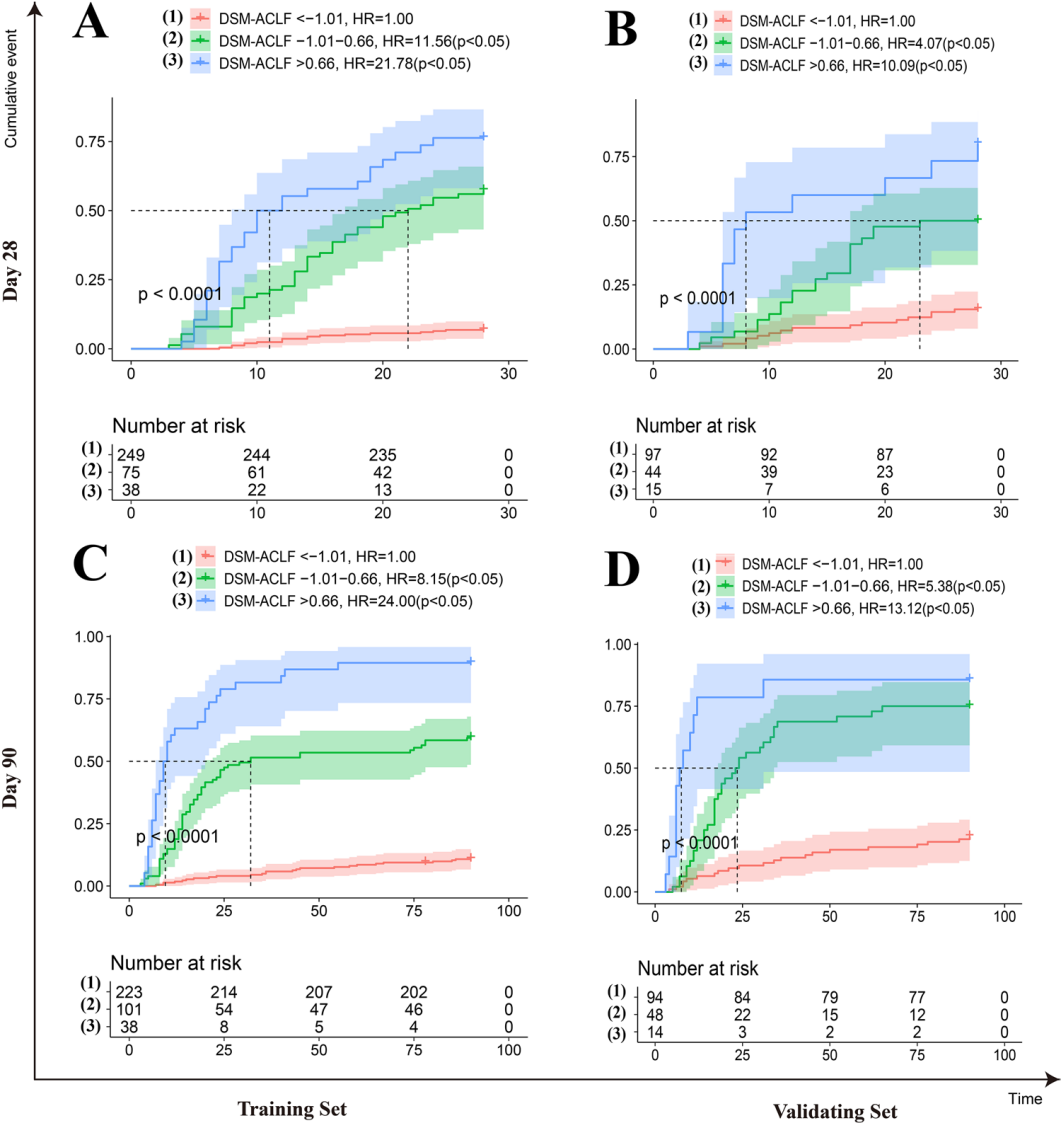
Risk stratification of the new score on day-1. (**A**,**B**) Risk stratification of the new score at 28-day since admission; (**A**,**C**) in the training set; (**C**,**D**) Risk stratification of the new score at 90-day since admission; (**B**,**D**) in the validation set.

Probability density function (PDF) curves were used to assess the predictive value of the different scoring models. For each scoring model, PDF curve results for 28-days and 90-days revealed the following overlap coefficients for the surviving and deceased groups in the training set: DSM-ACLF (day-1), 38.5% and 43.9%, respectively; COSSH-ACLF II, 45.6% and 53.8%; COSSH-ACLF, 51.7% and 55.1%; CLIF-C ACLF, 55.0% and 62.4%; MELD, 66.9% and 68.7%; MELD-Na, 67.7% and 69.2%. The DSM-ACLF (day-1) curve therefore showed the best discrimination for predicting the survival of HBV-ACLF patients. Further verification using the validation set also showed good discrimination (Fig. 3). Individual DSM-ACLF (day-1) patient scores from the training set were divided into low-risk (< − 1.01 points), medium-risk (− 1.01 to 0.66 points) and high-risk (> 0.66 points) groups. Compared to the low-risk group, the hazard ratios for death at 28-days and 90-days were significantly higher in the medium-risk (11.56 and 8.15, respectively) and high-risk (21.78 and 24.00) groups (Fig. 4).

### Changes in clinical features at day-3 of hospitalization

The clinical data for patients on day-3 are shown in Table 3. Following comprehensive medical treatment, 133 patients improved to non-ACLF and had significantly lower COSH-ACLF-II, COSSH-ACLF, CLIF-C ACLF, MELD and MELD-Na scores than the remaining ACLF patients. Moreover, the 28-day and 90-day liver transplant-free mortality rates for these non-ACLF patients were 7.5% and 15.8% respectively, which were also significantly lower than those of ACLF patients. Patients with different HBV-ACLF grades showed significant differences in AST, ALT, TB, total bile acid, glutamyl transferase, creatinine, triglyceride, total cholesterol, very low-density lipoprotein, monocytes, WBC, neutrophils, PT, INR, D-dimer and fibrin. The major differences in organ failures between ACLF grades 1, 2, and 3 were liver failure and coagulation failure. The incidence of liver failure for these grades was 75.11%, 79.66% and 80.95%, respectively, and all were lower than observed on day-1.

**Table 3:** Clinical characteristics of the enrolled patients on day-3.

Characteristics	Non-ACLF (n = 133)	ACLF-1 (n = 225)	ACLF-2 (n = 118)	ACLF-3 (n = 42)	*P*
Male	111 (83.46%)	193 (85.78%)	101 (85.59%)	35 (83.33%)	0.924
Age (years)	48.49 ± 12.79	47.72 ± 11.32	50.97 ± 11.65	51.4 ± 10.926	0.051
Vital signs					
MAP (mmHg)	87.67 (76.83,94.67)	90.00 (79.50,94.33)	91.17 (77.25,95.75)	87.83 (73.33,95.83)	0.753
SPO2 (%)	98.00 (98.00,99.00)	98.00 (97.50,99.00)	98.00 (97.00,99.00)	97.00 (96.50,98.00)	< 0.001
Complications					
Hepatic encephalopathy	0	5 (2.22%)	24 (20.34%)	37 (88.10%)	< 0.001
Hepatorenal syndrome	1 (0.75%)	3 (1.33%)	5 (8.48%)	14 (33.33%)	< 0.001
Infection	39 (29.32%)	76 (33.78%)	52 (42.37%)	22 (52.38%)	0.011
Gastrointestinal haemorrhage	6 (4.51%)	8 (3.56%)	11 (9.32%)	5 (11.91%)	0.046
Severity scores					
COSH-ACLF-IIs	5.49 (4.90,6.03)	5.61 (5.10,6.11)	6.38 (5.77,6.97)	6.85 (6.44,8.49)	< 0.001
COSSH-ACLFs	5.30 (4.91,5.74)	5.52 (5.13,5.93)	6.30 (5.82,6.79)	7.67 (6.59,9.03)	< 0.001
CLIF-C ACLFs	37.84 ± 6.03	39.12 ± 5.60	43.83 ± 6.96	50.18 ± 8.43	< 0.001
MELD	17.52 (15.59,20.26)	19.85 (17.74,21.95)	23.55 (21.47,27.54)	30.87 (24.95,43.96)	< 0.001
MELD-Na	18.68 (16.39,22.06)	20.83 (18.33,24.05)	24.86 (21.96,28.30)	29.90 (25.54,42.85)	< 0.001
Laboratory data					
Total protein (g/L)	56.80 (52.55,60.55)	56.80 (51.95,61.50)	56.90 (52.18,61.35)	55.55 (52.73,59.28)	0.759
Albumin (g/L)	31.70 (29.15,34.35)	31.10 (29.40,33.10)	31.00 (28.98,33.28)	31.85 (29.00,34.30)	0.281
Alanine aminotransferase (U/L)	98.00 (58.00,163.00)	126.00 (66.00,245.00)	108.00 (54.00,199.50)	157.00 (74.50,385.25)	0.003
Aspartate aminotransferase (U/L)	74.00 (50.50,116.50)	82.00 (56.50,129.00)	87.50 (59.00,149.25)	106.50 (78.75,228.00)	< 0.001
Alkaline phosphatase (U/L)	121.00 (97.00,146.50)	125.00 (102.50,150.00)	127.00 (105.75,150.25)	114.00 (94.25,135.25)	0.037
Total bile acid (μmol/L)	152.10 (114.15,175.00)	256.40 (198.20,351.95)	308.50 (224.25,370.45)	291.40 (201.20,353.13)	< 0.001
Total bilirubin (μmol/L)	243.60 (181.00,320.75)	291.30 (228.55,386.55)	308.50 (224.25,370.45)	431.40 (335.85,521.58)	< 0.001
Direct bilirubin (μmol/L)	185.80 (141.15,241.35)	224.10 (175.85,302.50)	244.95 (180.80,312.90)	310.45 (213.78,374.73)	< 0.001
Indirect bilirubin (μmol/L)	49.10 (35.00,70.95)	58.80 (37.80,89.40)	84.70 (56.80,108.75)	131.40 (69.13,196.10)	< 0.001
Glutamyl transferase (U/L)	75.00 (53.00,107.00)	72.00 (49.00,110.50)	59.00 (39.75,80.00)	55.50 (42.50,79.50)	< 0.001
Creatinine (μmol/L)	63.00 (52.00,75.41)	64.00 (54.50,75.82)	63.50 (52.00,77.00)	87.00 (55.00,196.25)	< 0.001
Serum urea (mmol/L)	4.85 (3.96,6.30)	4.35 (3.46,5.81)	5.15 (3.64,6.60)	5.68 (3.90,13.60)	0.003
Triglyceride (mmol/L)	1.57 (1.14,2.13)	1.31 (1.00,1.87)	0.97 (0.80,1.15)	1.05 (0.85,1.24)	< 0.001
Total cholesterol (mmol/L)	2.95 (2.24,3.58)	2.53 (2.02,3.33)	2.11 (1.52,2.62)	1.75 (1.13,2.67)	< 0.001
High density lipoprotein (mmol/L)	0.22 (0.14,0.40)	0.20 (0.14,0.33)	0.26 (0.17,0.34)	0.20 (0.13,0.29)	0.025
Low density lipoprotein (mmol/L)	1.07 (0.47,2.12)	1.00 (0.40,1.87)	1.06 (0.52,1.69)	0.85 (0.47,1.40)	0.559
Very low density lipoprotein (mmol/L)	1.22 (0.75,1.83)	1.08 (0.63,1.67)	0.63 (0.38,0.90)	0.60 (0.23,0.88)	< 0.001
Glucose (mmol/L)	5.33 (4.34,6.37)	4.96 (3.94,6.27)	4.75 (3.55,6.88)	4.01 (3.01,7.12)	0.030
K (mmol/L)	4.01 (3.72,4.41)	4.08 (3.75,4.46)	4.09 (3.65,4.44)	3.94 (3.60,4.83)	0.653
Na (mmol/L)	138.00 (136.00,140.00)	138.00 (136.00,140.00)	139.00 (135.00,141.00)	137.50 (135.00,141.00)	0.621
Cl (mmol/L)	102.00 (99.00,104.00)	102.00 (100.00,104.00)	102.00 (99.00,104.00)	99.50 (95.50,103.00)	0.038
Ca (mmol/L)	2.07 (1.98,2.18)	2.07 (1.99,2.15)	2.08 (1.99,2.15)	2.09 (1.99,2.19)	0.767
P (mmol/L)	0.92 (0.79,1.07)	0.93 (0.79,1.12)	0.87 (0.74,1.09)	1.10 (0.79,1.55)	0.018
White blood cell (10^9^/L)	6.70 (4.85,9.55)	7.60 (5.75,10.55)	7.75 (5.10,10.65)	10.55 (6.05,13.78)	0.001
Neutrophil (10^9^/L)	5.40 (3.50,7.65)	5.70 (3.60,8.55)	5.70 (3.65,8.40)	7.75 (4.73,11.10)	0.007
Lymphocyte (10^9^/L)	0.95 (0.70,11.30)	1.02 (0.75,1.48)	1.00 (0.66,1.38)	0.99 (0.70,1.16)	0.512
Monocyte (10^9^/L)	0.53 (0.39,0.74)	0.65 (0.44,0.87)	0.70 (0.44,1.05)	0.92 (0.56,1.43)	< 0.001
Eosinophil (10^9^/L)	0.02 (0.00,0.09)	0.02 (0.00,0.09)	0.03 (0.00,0.09)	0.03 (0.00,0.10)	0.926
Basophil (10^9^/L)	0.01 (0.01,0.03)	0.02 (0.01,0.03)	0.02 (0.01,0.03)	0.02 (0.01,0.03)	0.270
Red blood cell (10^12^/L)	3.58 (3.14,4.02)	3.65 (3.16,54.11)	3.43 (2.93,3.90)	3.46 (3.07,4.15)	0.122
Haemoglobin (g/L)	113.00 (101.00,125.00)	115.00 (103.00,129.00)	110.00 (97.75,124.00)	108.50 (95.25,132.25)	0.190
Haematocrit (%)	31.90 (27.85,35.55)	32.80 (29.40,36.50)	31.50 (27.45,35.58)	31.00 (27.98,36.30)	0.147
Platelet count (10^9^/L)	88.00 (57.00,130.00)	91.00 (60.50,130.00)	74.50 (52.50,107.25)	79.00 (49.50,113.00)	0.005
INR	1.55 (1.36,1.73)	1.70 (1.51,1.92)	2.30 (2.07,2.59)	3.04 (2.23,4.09)	< 0.001
Fibrin (g/L)	1.62 (1.34,1.98)	1.54 (1.22,1.87)	1.08 (0.85,1.37)	0.89 (0.50,1.12)	< 0.001
Prothrombin time (s)	17.70 (15.55,19.80)	19.50 (17.15,21.50)	25.50 (23.35,28.73)	33.95 (25.43,41.68)	< 0.001
D-Dimer (ug/L)	1696.00 (795.50,3317.00)	2295.00 (1017.00,4117.00)	4385.00 (2427.75,6862.25)	4472.00 (3247.50,7500.25)	< 0.001
Organ failures					
Liver	0	169 (75.11%)	94 (79.66%)	34 (80.95%)	0.016
Kidneys	0	3 (1.33%)	3 (2.54%)	15 (35.71%)	< 0.001
Coagulation	0	48 (21.33%)	114 (96.61%)	42 (100.00%)	< 0.001
Brain	0	5 (2.22%)	24 (20.34%)	37 (88.10%)	< 0.001
Circulation	0	0	0.85% (1)	6 (14.29%)	< 0.001
Lungs	0	0	0	2 (4.76%)	< 0.001
LT-free mortality					
28-day	10 (7.52%)	32 (14.22%)	59 (50.00%)	37 (88.10%)	< 0.001
90-day	21 (15.79%)	57 (25.33%)	72 (61.02%)	37 (88.10%)	< 0.001

Data are presented as the means ± SD, medians with (p25, p75), or numbers of patients (percentages).

ACLF, acute-on-chronic liver failure; MAP, Mean artery pressure; COSSH-ACLFs, COSSH-ACLF score; COSH-ACLF-IIs, COSSH-ACLF II score; CLIF-C ACLFs, CLIF Consortium ACLF score; MELD, Model for end-stage liver disease. LT, liver transplantation.

*P* value of comparisons across grades (ACLF-1, ACLF-2 and ACLF-3).

The incidence of coagulation failure on day-3 for ACLF grades 1, 2, and 3 was 21.33%, 96.61% and 100%, respectively, with all showing an increase compared to day-1. The incidence of organ failure on day-3 also increased with increasing ACLF grade (brain: 2.22%, 20.34%, 88.10%; kidney: 1.33%, 2.54%, 35.71%; circulation: 0%, 0.85%, 14.29%; respiratory: 0%, 0%, 4.72%). Day-3 clinical data for surviving and deceased HBV-ACLF patients is shown in Table 4. The incidence of HE, hepatorenal syndrome, infection and GIH was significantly higher in deceased patients compared to survivors, as were the COSH-ACLF-II, COSSH-ACLF, CLIF-C ACLF, MELD, and MELD-Na scores. The surviving and deceased patient groups also showed significant differences in TB, IB, glutamyl transferase, serum urea, triglycerides, total cholesterol, very low-density lipoprotein, neutrophils, lymphocytes, platelet count, PT, INR, D-dimer and fibrin (all *P* < 0.05).

**Table 4:** Comparison of variable characteristics between the survival group and death groups on day-3.

Characteristics	28-day survival group (n = 380)	28-day death group (n = 138)	*P*	90-day survival group (n = 331)	90-day death group (n = 187)	*P*
Male	331 (87.11%)	109 (78.99%)	0.022	292 (88.22%)	148 (79.14%)	0.006
Age (years)	46.99 ± 11.54	54.38 ± 10.85	< 0.001	46.00 (38.00,55.00)	54.00 (47.00,60.00)	< 0.001
Therapies						
Vasopressors	0	7 (5.07%)	< 0.001	0	7 (3.74%)	< 0.001
Mechanical ventilation	0	2 (1.45%)	0.019	0	2 (1.07%)	0.059
Complications						
Hepatic encephalopathy	3.42% (13)	38.41% (53)	< 0.001	3.32% (11)	29.41% (55)	< 0.001
Hepatorenal syndrome	1.32% (5)	13.04% (18)	< 0.001	1.21% (4)	10.16% (19)	< 0.001
Infection	33.42% (127)	44.93% (62)	0.016	32.33% (107)	43.85% (82)	0.009
Gastrointestinal haemorrhage	3.68% (14)	11.59% (16)	< 0.001	2.42% (8)	11.76% (22)	< 0.001
Severity scores						
COSH-ACLF-IIs	5.56 (5.04,6.03)	6.76 (6.30,7.32)	< 0.001	5.50 (4.98,5.93)	6.55 (6.04,7.19)	< 0.001
COSSH-ACLFs	5.48 (5.10,5.91)	6.61 (6.10,7.49)	< 0.001	5.39 (5.04,5.82)	6.34 (5.84,7.21)	< 0.001
CLIF-C ACLFs	38.56 ± 5.60	46.83 ± 7.73	< 0.001	38.29 ± 5.61	45.14 ± 7.68	< 0.001
MELD	19.61 (16.97,22.12)	24.39 (20.55,30.93)	< 0.001	19.13 (16.50,21.64)	23.58 (20.30,28.66)	< 0.001
MELD-Na	20.65 (17.84,23.60)	26.08 (21.02,31.51)	< 0.001	20.41 (17.55,23.24)	24.98 (20.84,29.11)	< 0.001
Vital signs						
MAP (mmHg)	89.33 (78.00,94.67)	89.83 (77.67,95.75)	0.545	89.33 (78.67,94.67)	89.67 (76.67,95.67)	0.935
SPO2 (%)	98.00 (98.00,99.00)	98.00 (97.00,99.00)	0.002	98.00 (98.00,99.00)	98.00 (97.00,99.00)	0.003
Laboratory data						
Total protein (g/L)	56.80 (52.80,61.18)	56.15 (51.30,61.23)	0.226	57.00 (52.80,61.40)	56.20 (51.70,60.30)	0.106
Albumin (g/L)	31.30 (29.23,33.80)	31.20 (28.90,33.83)	0.734	31.30 (29.20,33.80)	31.10 (28.90,33.80)	0.236
Globulin (g/L)	25.00 (21.93,29.20)	24.35 (19.48,29.40)	0.121	25.10 (21.90,29.20)	24.40 (20.50,29.30)	0.743
Alanine aminotransferase (U/L)	110.50 (61.00,205.50)	118.00 (68.50,262.25)	0.229	114.00 (61.00,212.00)	111.00 (64.00,248.00)	0.743
Aspartate aminotransferase (U/L)	81.00 (54.00,124.00)	99.50 (66.50,177.25)	< 0.001	80.00 (53.00,120.00)	98.00 (62.00,168.00)	< 0.001
Alkaline phosphatase (U/L)	125.00 (102.25,148.00)	125.00 (100.00,148.00)	0.595	126.00 (103.00,149.00)	123.00 (99.00,147.00)	0.295
Total bile acid (μmol/L)	227.29 (157.53,315.50)	247.60 (158.35,338.25)	0.382	220.70 (158.00,315.70)	247.60 (156.40,332.00)	0.398
Total bilirubin (μmol/L)	265.35 (202.60,360.30)	379.50 (295.58,480.20)	< 0.001	258.50 (196.20,339.00)	372.50 (293.30,477.30)	< 0.001
Direct bilirubin (μmol/L)	209.00 (159.53,274.18)	275.85 (204.60,362.70)	< 0.001	198.10 (152.00,261.30)	268.00 (213.30,355.20)	< 0.001
Indirect bilirubin (μmol/L)	57.40 (36.85,86.43)	90.20 (56.65,136.00)	< 0.001	52.90 (35.00,81.50)	86.80 (59.20,126.70)	< 0.001
Glutamyl transferase (U/L)	71.00 (50.25,103.00)	57.50 (37.75,89.50)	< 0.001	72.00 (51.00,102.00)	60.00 (40.00,94.00)	< 0.001
Creatinine (μmol/L)	64.00 (54.00,75.00)	67.00 (52.00,93.50)	0.048	64.00 (54.00,74.00)	67.00(52.00,86.00)	0.076
Serum urea (mmol/L)	4.53 (3.61,5.74)	5.79 (4.08,9.65)	< 0.001	4.43 (3.56,5.65)	5.45 (4.10,8.27)	< 0.001
Triglyceride (mmol/L)	1.28 (1.00,1.83)	1.02 (0.85,1.33)	< 0.001	1.31 (1.01,1.88)	1.06 (0.88,1.37)	< 0.001
Total cholesterol (mmol/L)	2.56 (2.01,3.36)	2.16 (1.57,2.69)	< 0.001	2.57 (2.04,3.39)	2.24 (1.52,2.83)	< 0.001
High density lipoprotein (mmol/L)	0.21 (0.14,0.34)	0.25 (0.16,0.36)	0.136	0.21 (0.15,0.34)	0.23 (0.15,0.35)	0.391
Low density lipoprotein (mmol/L)	1.01 (0.41,1.87)	1.02 (0.54,1.59)	0.683	1.01 (0.44,1.87)	0.96 (0.45,1.69)	0.455
Very low density lipoprotein (mmol/L)	1.05 (0.63,1.64)	0.65 (0.32,1.07)	< 0.001	1.09 (0.65,1.67)	0.69 (0.34,1.16)	< 0.001
Glucose (mmol/L)	4.85 (3.90,6.16)	5.22 (3.94,7.85)	0.034	4.84 (3.91,6.13)	5.21 (3.92,7.77)	0.011
K (mmol/L)	4.05 (3.75,4.44)	4.04 (3.58,4.50)	0.604	4.09 (3.81,4.44)	3.99 (3.58,4.49)	0.118
Na (mmol/L)	138.00 (136.00,140.00)	138.50 (135.00,140.00)	0.615	138.00 (136.00,140.00)	138.00 (135.00,140.00)	0.951
Cl (mmol/L)	102.00 (100.00,104.00)	101.00 (98.00,104.00)	0.033	102.00 (100.00,104.00)	101.00 (98.00,104.00)	0.004
Ca (mmol/L)	2.08 (1.99,2.15)	2.08 (1.99,2.18)	0.265	2.07 (1.99,2.15)	2.08 (1.98,2.18)	0.314
P (mmol/L)	0.93 (0.80,1.10)	0.89 (0.72,1.14)	0.302	0.95 (0.81,1.11)	0.88 (0.71,1.11)	0.034
White blood cell (10^9^/L)	7.20 (5.20,9.70)	9.00 (6.20,11.93)	< 0.001	7.10 (5.20,9.80)	8.60 (6.10,11.30)	0.003
Neutrophil (10^9^/L)	5.30 (3.50,7.90)	6.95 (4.50,9.90)	< 0.001	5.30 (3.50,7.93)	6.50 (4.30,9.30)	< 0.001
Lymphocyte (10^9^/L)	1.05 (0.76,1.47)	0.87 (0.63,1.18)	< 0.001	1.08 (0.77,1.48)	0.86 (0.62,1.23)	< 0.001
Monocyte (10^9^/L)	0.60 (0.41,0.83)	0.76 (0.48,1.11)	< 0.001	0.61 (0.42,0.83)	0.71 (0.43,1.06)	0.024
Eosinophil (10^9^/L)	0.03 (0.00,0.09)	0.01 (0.00,0.06)	0.027	0.03 (0.00,0.09)	0.01 (0.00,0.07)	0.023
Basophil (10^9^/L)	0.02 (0.01,0.03)	0.01 (0.01,0.03)	0.051	0.02 (0.01,0.03)	0.01 (0.01,0.03)	0.008
Red blood cell (10^12^/L)	3.63 (3.18,4.10)	3.35 (2.95,3.90)	< 0.001	3.64 (3.19,4.08)	3.38 (2.97,3.92)	0.002
Haemoglobin (g/L)	115.00 (103.00,128.00)	108.00 (93.00,124.25)	0.003	115.00 (103.00,128.00)	109.00 (96.00,125.00)	0.004
Haematocrit (%)	32.45 (29.13,36.30)	31.15 (27.58,35.13)	0.022	32.50 (29.30,36.30)	31.10 (27.50,35.20)	0.008
Platelet count (10^9^/L)	88.00 (61.00,126.00)	73.50 (46.00,113.00)	< 0.001	91.00 (61.00,130.00)	71.00 (48.00,111.00)	< 0.001
INR	1.69 (1.47,1.95)	2.30 (1.98,3.01)	< 0.001	1.65 (1.45,1.91)	2.16 (1.89,2.72)	< 0.001
Fibrin (g/L)	1.51 (1.17,1.87)	1.11 (0.83,1.49)	< 0.001	1.51 (1.17,1.87)	1.20 (0.87,1.56)	< 0.001
Prothrombin time (s)	19.20 (17.00,21.90)	25.90 (22.40,32.65)	< 0.001	18.80 (16.70,21.50)	24.50 (21.30,30.40)	< 0.001
D-Dimer (ug/L)	2079.00 (951.25,3876.00)	4484.00 (2946.50,7198.75)	< 0.001	2006.00 (856.00,3749.00)	4220.00 (2404.00,7001.00)	< 0.001

Data are presented as the means ± SD, medians with (p25, p75), or numbers of patients (percentages).

ACLF, acute-on-chronic liver failure; MAP, Mean artery pressure; COSSH-ACLFs, COSSH-ACLF score; COSH-ACLF-IIs, COSSH-ACLF II score; CLIF-C ACLFs, CLIF Consortium ACLF score; MELD, Model for end-stage liver disease. LT, liver transplantation.

### Development and validation of a new prognostic score based on day-3 clinical data

Using 28-day death as the outcome variable, the LASSO algorithm was used to screen 10 variables in the training set: age, HE, ALP, TB, TG, VLDL, blood glucose, neutrophils, fibrin and PT. Logistic regression modelling was used to construct the DSM-ACLF (day-3) score for HBV-ACLF patients as follows: DSM-ACLF (day-3) = −9.780 $+$ 0.072 $\times$ age $+$ 1.872 $\times$ HE $+$ 0.008 $\times$ ALP $+$ 0.005 $\times$ TB $-$ 0.572 $\times$ TG $-$ 0.190 $\times$ VLDLC $+$ 0.102 $\times$ GLU $+$ 0.145 $\times \text{neutrophils}-$ 0.628 $\times$ Fib $+$ 0.098 $\times$ PT.

**Figure 5:**
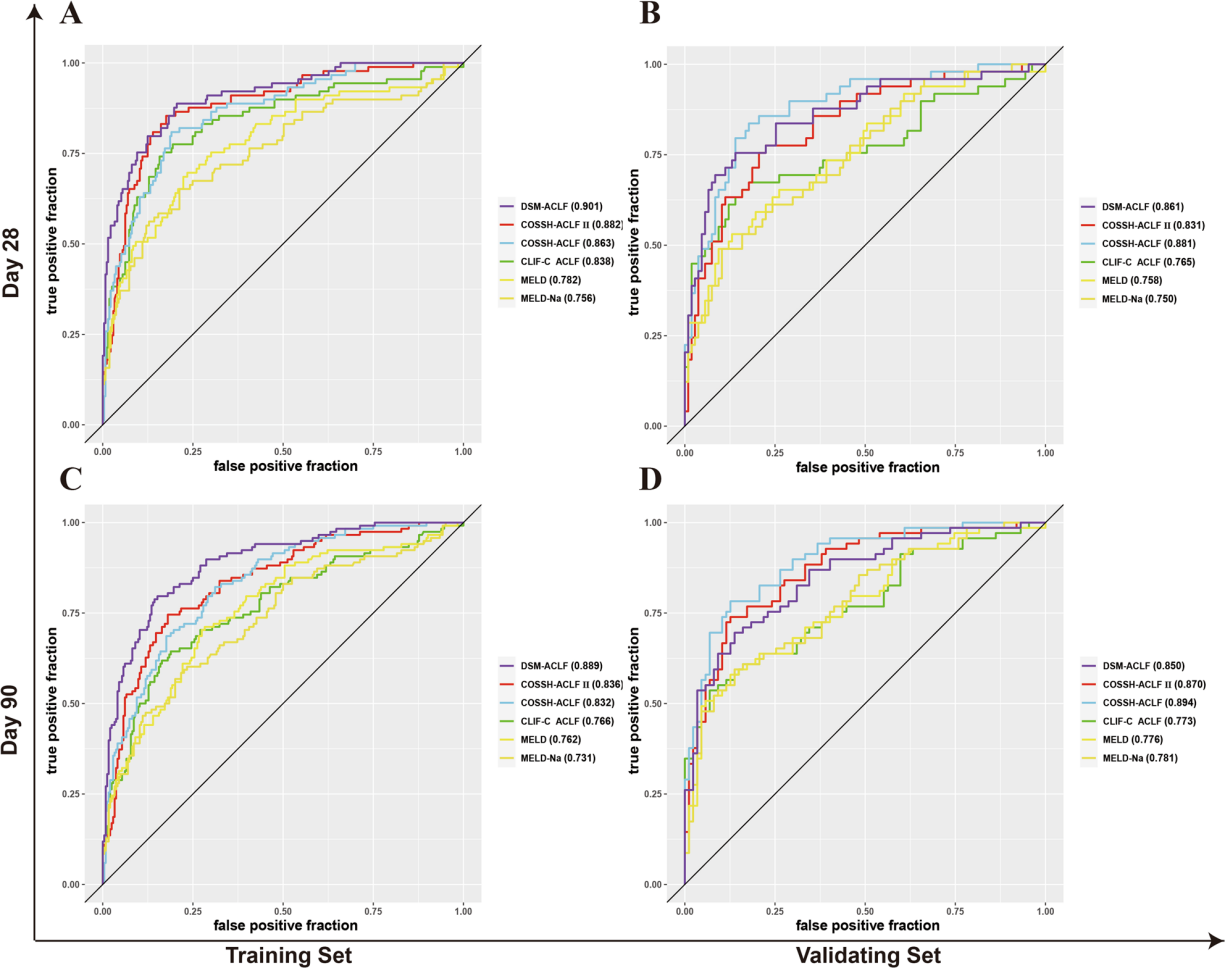
Time-dependent ROC curves of the new score and of the five other scores on day-3. (**A**,**B**) time-dependent ROC curves at 28 day since admission; (**A**,**C**) in the training set; (**C**,**D**) time-dependent ROC curves at 90 day since admission; (**B**,**D**) in the validation set.

The AUCs obtained from ROC analysis of the training set for 28-days and 90-days were as follows: DSM-ACLF (day-3), 0.901 and 0.889, respectively; COSSH-ACLF II, 0.882 and 0.836; COSSH-ACLF, 0.863 and 0.832; CLIF-C ACLF, 0.838 and 0.766; MELD, 0.782 and 0.762; MELD-Na, 0.756 and 0.731. The AUCs for the DSM-ACLF (day-3) score were both close to 0.9, and higher than those of the other five scoring systems, indicating better predictive value. In the validation dataset, the new DSM-ACLF (day-3) scoring model showed AUCs from ROC analysis for 28-days and 90-days of 0.861 and 0.850, respectively (Fig. 5). Calibration curves and decision curves were then plotted for all six prognostic scoring systems. As shown in Supplementary Fig. 2, the DSM-ACLF (day-3) prognostic score had better calibration and net gain than the other prognostic scoring systems in both the training and validation sets.

**Figure 6:**
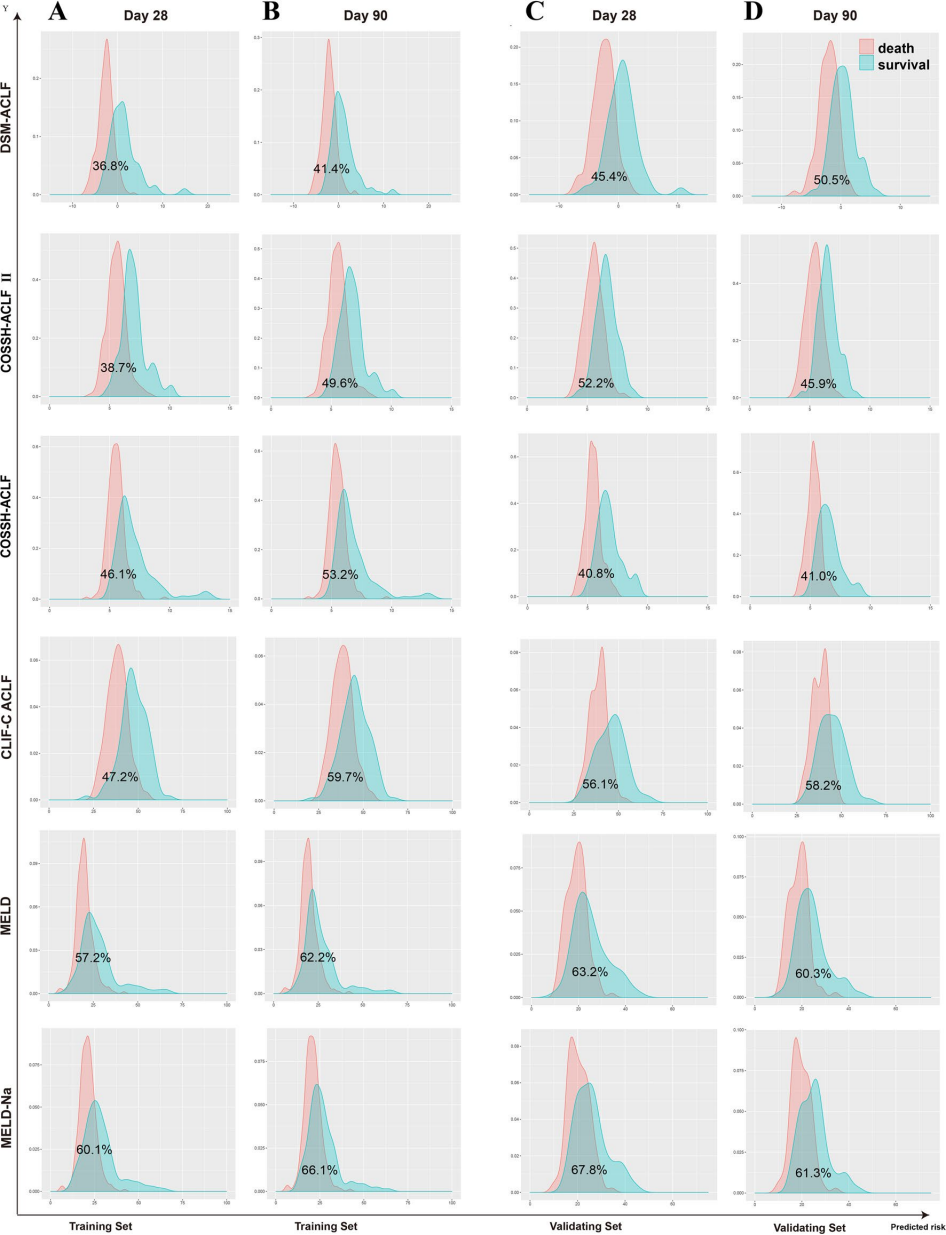
Probability density function (PDF) of the new score and of the five other scores on day-3. (**A**,**B**) in the training set; time-dependent ROC curves at 28 day since admission; (**A**,**C**) PDF at 28 day since admission; (**C**,**D**) in the validation set; (**B**,**D**) PDF at 90 day since admission.

**Figure 7:**
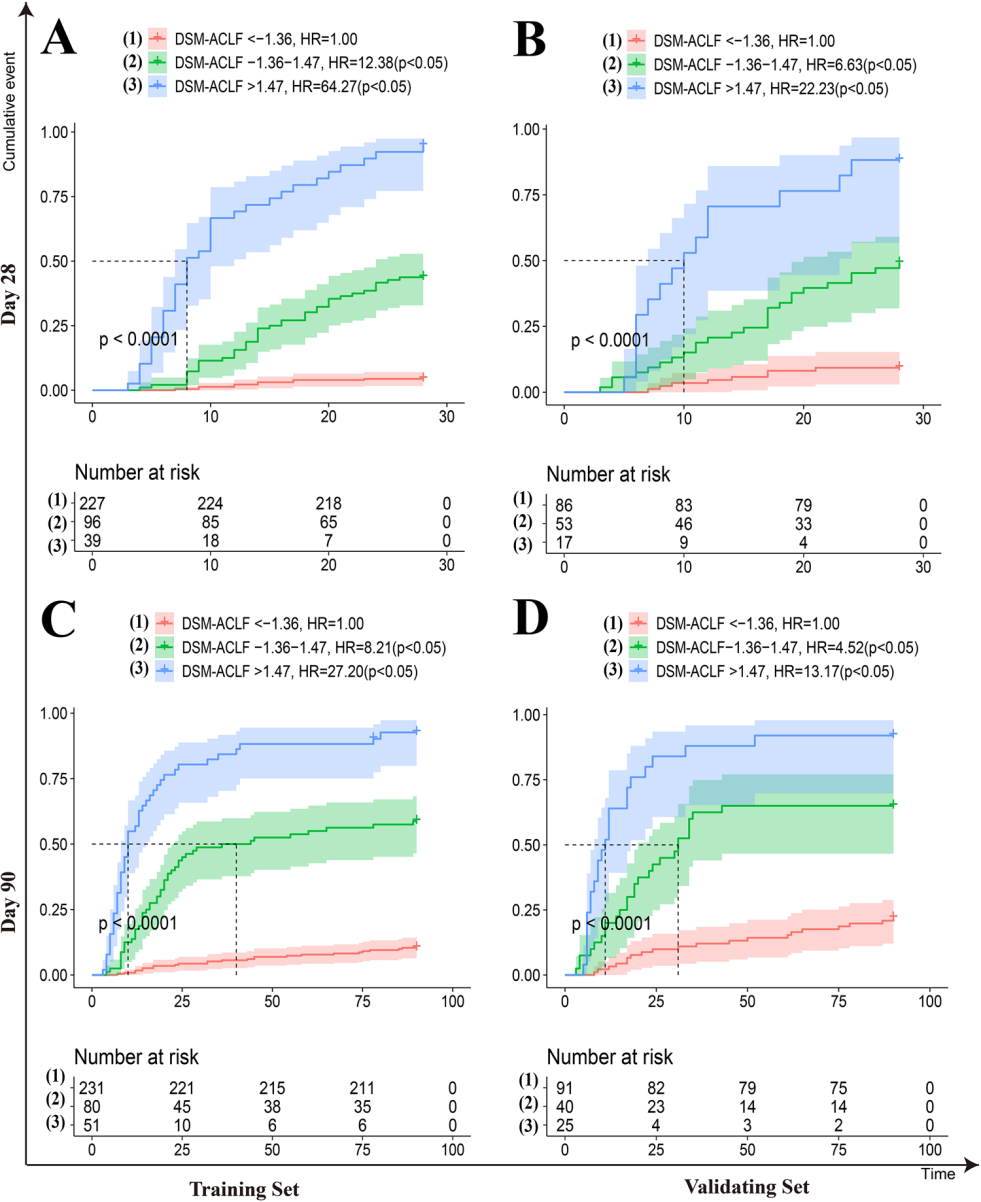
Risk stratification of the new score on day-3. (**A**,**B**) Risk stratification of the new score at 28-day since admission; (**A**,**C**) in the training set; (**C**,**D**) Risk stratification of the new score at 90-day since admission; (**B**,**D**) in the validation set.

In the training set, the overlap coefficients for the DSM-ACLF (day-3) score at 28-days and 90-days were 36.8% and 41.4%, respectively. These were both lower than those of the other five prognostic models. In the validation set, the DSM-ACLF (day-3) score maintained a good discrimination, with 28-day and 90-day overlap coefficients of 45.4% and 50.5%, respectively (Fig. 6). DSM-ACLF (day-3) scores for the training set were divided into low-risk (< -1.36 points), intermediate-risk (− 1.36 to 1.47 points) and high-risk (> 1.47 points) groups. The 28-day and 90-day hazard ratios for the intermediate-risk (12.38 and 8.21, respectively) and high-risk (64.27 and 27.20) groups were significantly higher than those of the low-risk group. The DSM-ACLF (day-3) score also showed good prognostic risk stratification in the validation set (Fig. 7).

### Final optimization of the novel prognostic score model for HBV-ACLF patients

The day-1 and day-3 data were overlaid to construct the 1 + 3-day prognostic score model (Supplementary Tables and Figures). A pooled comparison of the AUC was then performed for the prognostic scoring models with day-1, day-3, and day-(1 + 3) data (Table 4). This analysis revealed that 8 models had predictive value for the outcome of HBV-ACLF patients: DSM-ACLF (day-1), DSM-ACLF (day-3), DSM-ACLF (day-1 + 3), COSH-ACLF-II, COSSH-ACLF, CLIF-C ACLF, MELD and MELD-Na. The DSM-ACLF (day-3) model showed the highest predictive value, with AUCs of 0.901 and 0.899 at 28-days and 90-days, respectively. Overall, the above results demonstrate that dynamic monitoring of clinical changes can significantly improve the prognostic accuracy of each scoring model (Table 5).

**Table 5:** Prognostic value of each scoring model on day-1, day-3 and day-(1 + 3) in HBV-ACLF patients.

Model	Time	Training set				Validation set			
		day mortality AUC (95%CI)	*P*	90-day mortality AUC (95%CI)	*P*	28-day mortality AUC (95%CI)	*P*	90-day mortality AUC (95%CI)	*P*
DSM-ACLF	Day-1	**0.879(0.837–0.922)**		**0.857(0.814–0.900)**		**0.805(0.729–0.881)**		**0.799(0.729–0.870)**	
COSH-ACLF IIs		0.857(0.814–0.901)	0.141	0.818(0.771–0.864)	0.018	0.762(0.680–0.844)	0.146	0.789(0.717–0.860)	0.714
COSSH-ACLFs		0.825(0.775–0.875)	0.009	0.811(0.763–0.859)	0.005	0.781(0.701–0.860)	0.432	0.783(0.711–0.856)	0.556
CLIF-C ACLFs		0.805(0.752–0.859)	0.001	0.760(0.706–0.814)	0.001	0.683(0.585–0.780)	0.002	0.724(0.642–0.806)	0.051
MELD		0.703(0.634–0.772)	0.001	0.712(0.653–0.771)	0.001	0.629(0.528–0.730)	0.001	0.629(0.537–0.720)	0.001
MELD-Na		0.703(0.635–0.770)	0.001	0.706(0.647–0.765)	0.001	0.609(0.507–0.711)	0.001	0.621(0.530–0.712)	0.001
DSM-ACLF	Day-3	**0.901(0.871–0.944)**		**0.889(0.853–0.924)**		**0.861(0.794–0.928)**		**0.850(0.790–0.911)**	
COSH-ACLF IIs		0.882(0.841–0.924)	0.095	0.836(0.791–0.880)	0.004	0.831(0.762–0.901)	0.234	0.870(0.814–0.925)	0.361
COSSH-ACLFs		0.863(0.819–0.906)	0.011	0.832(0.789–0.875)	0.001	0.881(0.824–0.939)	0.368	0.894(0.845–0.943)	0.035
CLIF-C ACLFs		0.838(0.785–0.891)	0.001	0.766(0.711–0.821)	0.001	0.765(0.675–0.856)	0.003	0.773(0.697–0.849)	0.013
MELD		0.782(0.722–0.842)	0.001	0.762(0.708–0.815)	0.001	0.758(0.674–0.841)	0.025	0.776(0.701–0.850)	0.001
MELD-Na		0.756(0.692–0.820)	0.001	0.731(0.674–0.789)	0.001	0.750(0.668–0.833)	0.021	0.781(0.709–0.854)	0.001
DSM-ACLF	Day-(1 + 3)	**0.833(0.779–0.887)**		**0.890(0.854–0.926)**		**0.800(0.715–0.886)**		**0.859(0.799–0.919)**	
COSH-ACLF IIs		0.886(0.846–0.926)	0.009	0.839(0.795–0.883)	0.004	0.816(0.742–0.889)	0.701	0.850(0.789–0.910)	0.713
COSSH-ACLFs		0.868(0.825–0.912)	0.084	0.843(0.801–0.885)	0.002	0.864(0.801–0.928)	0.085	0.875(0.820–0.930)	0.456
CLIF-C ACLFs		0.835(0.782–0.887)	0.947	0.774(0.720–0.828)	0.001	0.738(0.643–0.834)	0.185	0.763(0.685–0.841)	0.008
MELD		0.768(0.706–0.829)	0.062	0.758(0.704–0.812)	0.001	0.724(0.633–0.815)	0.195	0.733(0.652–0.815)	0.001
MELD-Na		0.756(0.693–0.819)	0.033	0.740(0.684–0.796)	0.001	0.706(0.613–0.799)	0.122	0.734(0.652–0.815)	0.003

DSM-ACLF is the prognostic scoring model that we built based on dynamic data; AUC, area under curve. ACLF, acute-on-chronic liver failure; COSSH-ACLFs, COSSH-ACLF score; COSH-ACLF-IIs, COSSH-ACLF II score; CLIF-C ACLFs, CLIF Consortium ACLF score. MELD, Model for end-stage liver disease.

*P* value for comparisons between the AUC of DSM-ACLF and another model.

Significant values are in bold.

## Discussion

ACLF has become an active area of research due to its rapid onset, rapid progression and poor prognosis. Prognostic scoring models in particular have been continuously investigated and updated by scholars^16^. Current scoring models are mostly based on electronic clinical data entered at the day of admission, even though the development of ACLF is a dynamic process. Therefore, an ACLF scoring model based on dynamic patient data could more accurately assess the likely progress of disease and thus provide a firm basis for subsequent treatment plans. Gustotet al. evaluated the clinical course of ACLF by comparing CLIF-C ACLF scores obtained at different time-points^9^. These authors found that prognosis was closely related to changes in the clinical status, and that clinical decisions could be adjusted using dynamic scores. Due to the rapidly changing course of HBV-ACLF, both the initial characteristics and the dynamic trends of clinical indicators are helpful in predicting the outcome of ACLF^10^. Evaluation at multiple time-points can more accurately reflect the clinical course and response to drug therapy, thereby improving the predictive power of the ACLF^17^.

Currently, five prognostic scoring models are commonly used for HBV-ACLF: COSSH-ACLF-II, COSSH-ACLF, CLIF-C ACLF, MELD and MELD-Na. Although these have predictive value for HBV-ACLF patients, all have certain shortcomings. The MELD score developed in 2000 does not take into account the impact of clinical complications such as hepatorenal syndrome, ascites, and HE on the outcome of HBV-ACLF patients^8,18^. MELD-Na was derived from the MELD score^19^, but some authors have suggested these models are not sufficiently sensitive or specific for the prediction of short-term outcomes in HBV-ACLF patients^20,21^. The CLIF-C ACLF scoring model includes the assessment of multiple organs and incorporates age and white blood cell count into the score^22^. This scoring system appears to be more accurate in evaluating the prognosis of ACLF patients than MELD and MELD-Na^23^. However, the CLIF-C ACLF score was developed based on data from a Western population with predominantly chronic hepatitis C and alcoholic liver disease. COSSH-ACLF^22^ and COSH-ACLF II^7^ are prognostic models aimed at the CHB population and take into account the evaluation of clinical indicators for multiple organ failure. Their performance is better than that of CLIF-C ACLF, MELD and MELD-Na. However, COSSH-ACLF and COSH-ACLF II focus only on the baseline indicators at admission and do not take into account the importance of dynamic changes in the patients' clinical characteristics.

In the present study, a novel prognostic scoring model based on dynamic time points was constructed. DSM-ACLF was developed based on clinical data from day-1 and day-3, as well as data from day-1 combined with day-3. Eight scoring models were found to have prognostic value for the outcome of HBV-ACLF patients: DSM-ACLF (day-1), DSM-ACLF (day-3), DSM-ACLF (day-1 + day-3), COSH-ACLF-II, COSSH-ACLF, CLIF-C ACLF, MELD and MELD-Na. Of these, DSM-ACLF (day-3) had the best performance, with AUCs of 0.901 and 0.889 for 28-day and 90-day follow-up, respectively. The calibration and clinical decision analysis of DSM-ACLF (day-3) maintained good performance. PDF analysis revealed the overlap coefficient for DSM-ACLF (day-3) scores in the surviving and deceased patient groups was the smallest and had the best prognostic discrimination. The DSM-ACLF (day-3) scoring system also allowed accurate stratification, with scores < − 1.361 classified as low-risk, − 1.36 to 1.47 classified as medium-risk group, and > 1.47 classified as high-risk. The risk stratification provided by the DSM-ACLF (day-3) model allows the severity of HBV-ACLF to be predicted with accuracy, and individualized treatment plans to be formulated.

The DSM-ACLF (day-3) score is easy to calculate and consists of age, HE, alkaline phosphatase, total bilirubin, triglycerides, very low-density lipoprotein, blood glucose, neutrophil, fibrin and PT. Among these factors, age is closely related to the severity of liver disease and poor prognosis^24^. Other studies have reported that age, PT, total bilirubin, infection, and hepatic encephalopathy are also independent factors for the prognosis of ACLF^25,26^. The DSM-ACLF (day-3) score includes the factors of blood glucose, triglycerides, very low-density lipoprotein and fibrin. These partly reflect the metabolic function of the liver, especially lipid metabolism. Hepatocytes and their microenvironment are damaged during liver failure, leading to dysfunction in liver metabolism, synthesis, and detoxification. Lipid metabolism disorders are thought to play an important role in the development of ACLF^27–29^. Xiao et al.^30^ suggest that many diseases can be attributed to defects in complex lipid metabolism. Lipids are ubiquitous in human biology and play roles in numerous intracellular and intercellular processes. These include non-lysosomal sphingolipid metabolism, acylceramide metabolism, de novo phospholipid synthesis, phospholipid remodeling, phosphatidylinositol metabolism, mitochondrial cardiolipin synthesis and remodeling, ether lipid metabolism, and the common clinical phenotypes associated with them. In this respect, the DSM-ACLF (day-3) score is the first prognostic score to introduce relevant indicators of lipid metabolism. An increasing number of studies have confirmed that timely intervention for lipid metabolism disorders of the liver not only meets the energy needs of the human body, but also promotes the repair and regeneration of damaged hepatocytes, enhances the liver's resistance to infection and toxins, and helps in the treatment of ACLF^31–33^.

In summary, we have developed a novel scoring system for HBV-ACLF referred to as DSM-ACLF (day-3). This is based on 10 factors that can accurately predict outcome and stratify the risk for short-term mortality in patients with HBV-ACLF.

### Supplementary Information

Supplementary Figure 1.

Supplementary Figure 2.

Supplementary Figure 3.

Supplementary Figure 4.

Supplementary Figure 5.

Supplementary Figure 6.

Supplementary Legends.

Supplementary Table 1.

Supplementary Table 2.

## Data Availability

The datasets used and/or analyzed during the current study available from the corresponding author on reasonable request.
