# Supplementary figures and images for: Construction of a novel prognostic scoring model for HBV-ACLF liver failure based on dynamic data
Source: Sci Rep. 2024 Jul 2;14:15198. doi: 10.1038/s41598-024-63900-4 (PMC11219721; doi:10.1038/s41598-024-63900-4)

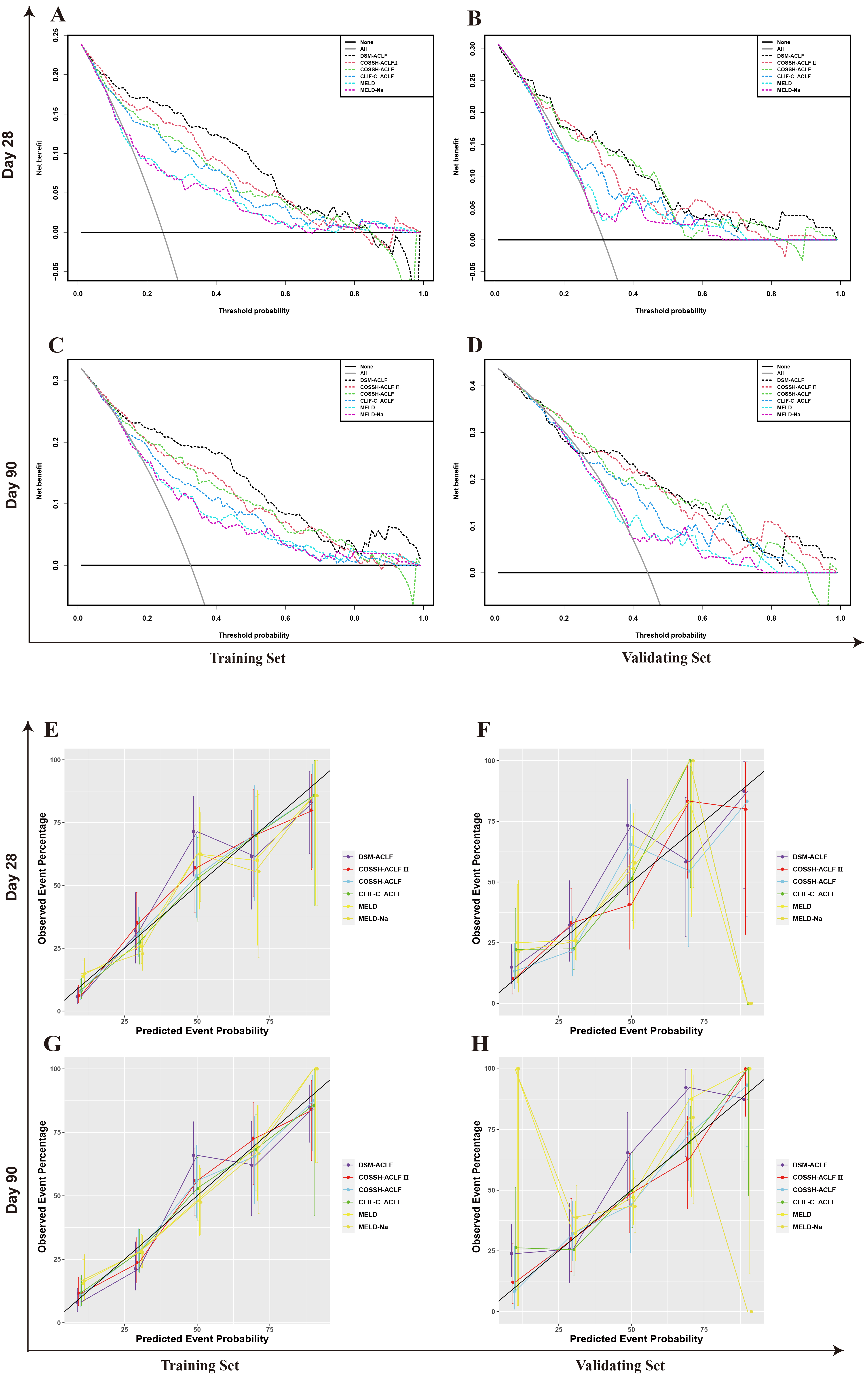

Supplement: Supplementary file 1 — Supplementary Figure 1. [file 41598_2024_63900_MOESM1_ESM.jpg]

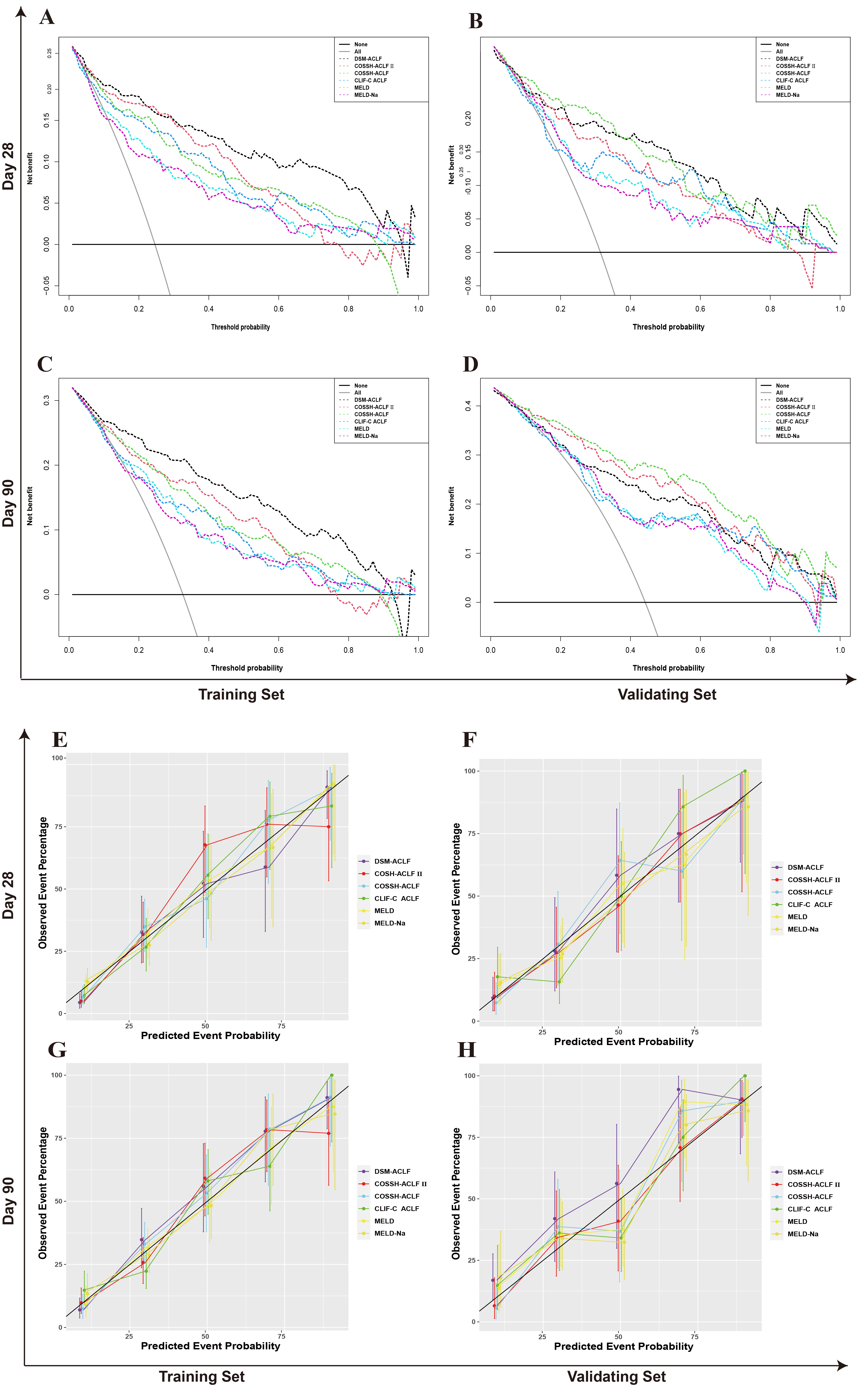

Supplement: Supplementary file 2 — Supplementary Figure 2. [file 41598_2024_63900_MOESM2_ESM.jpg]

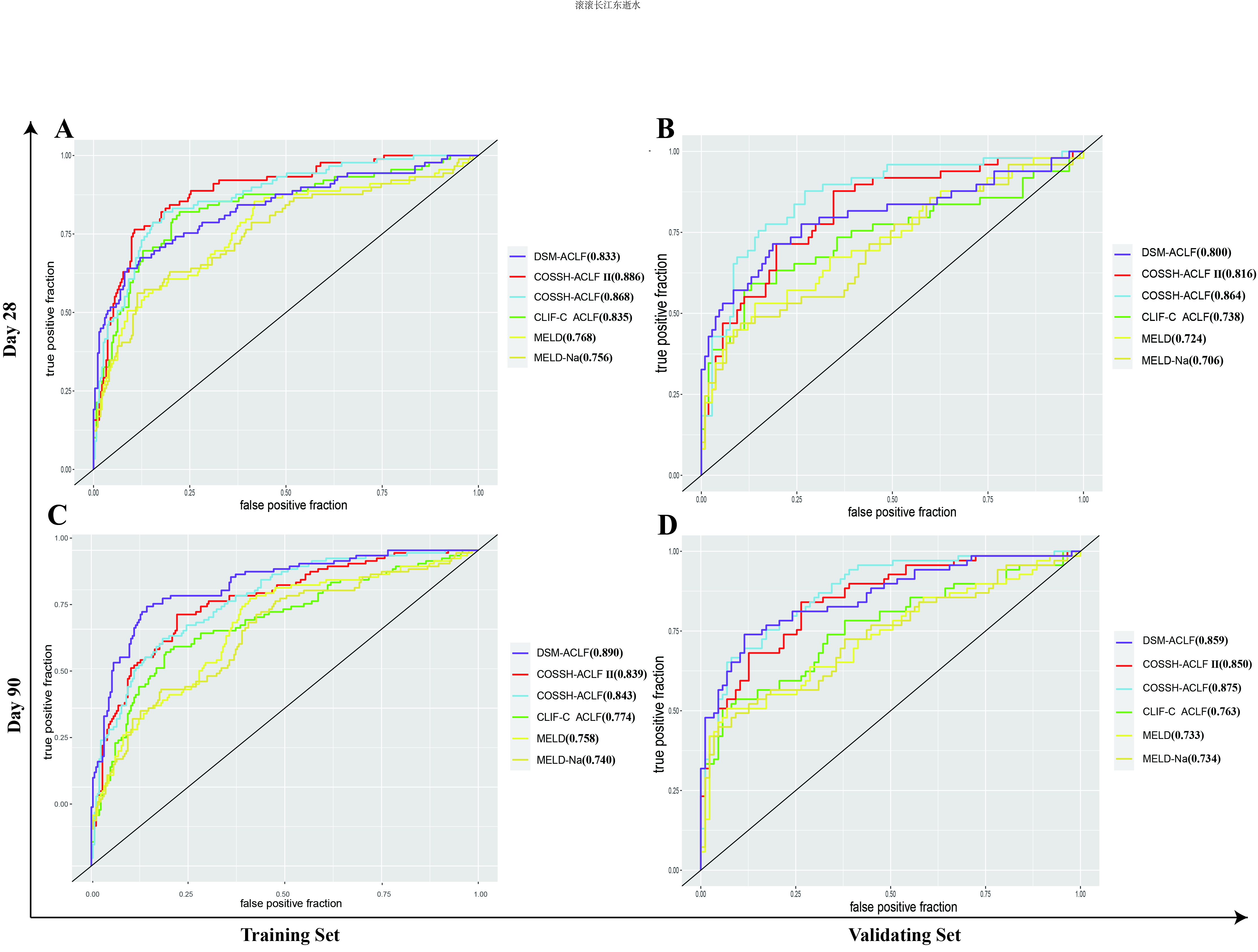

Supplement: Supplementary file 3 — Supplementary Figure 3. [file 41598_2024_63900_MOESM3_ESM.jpg]

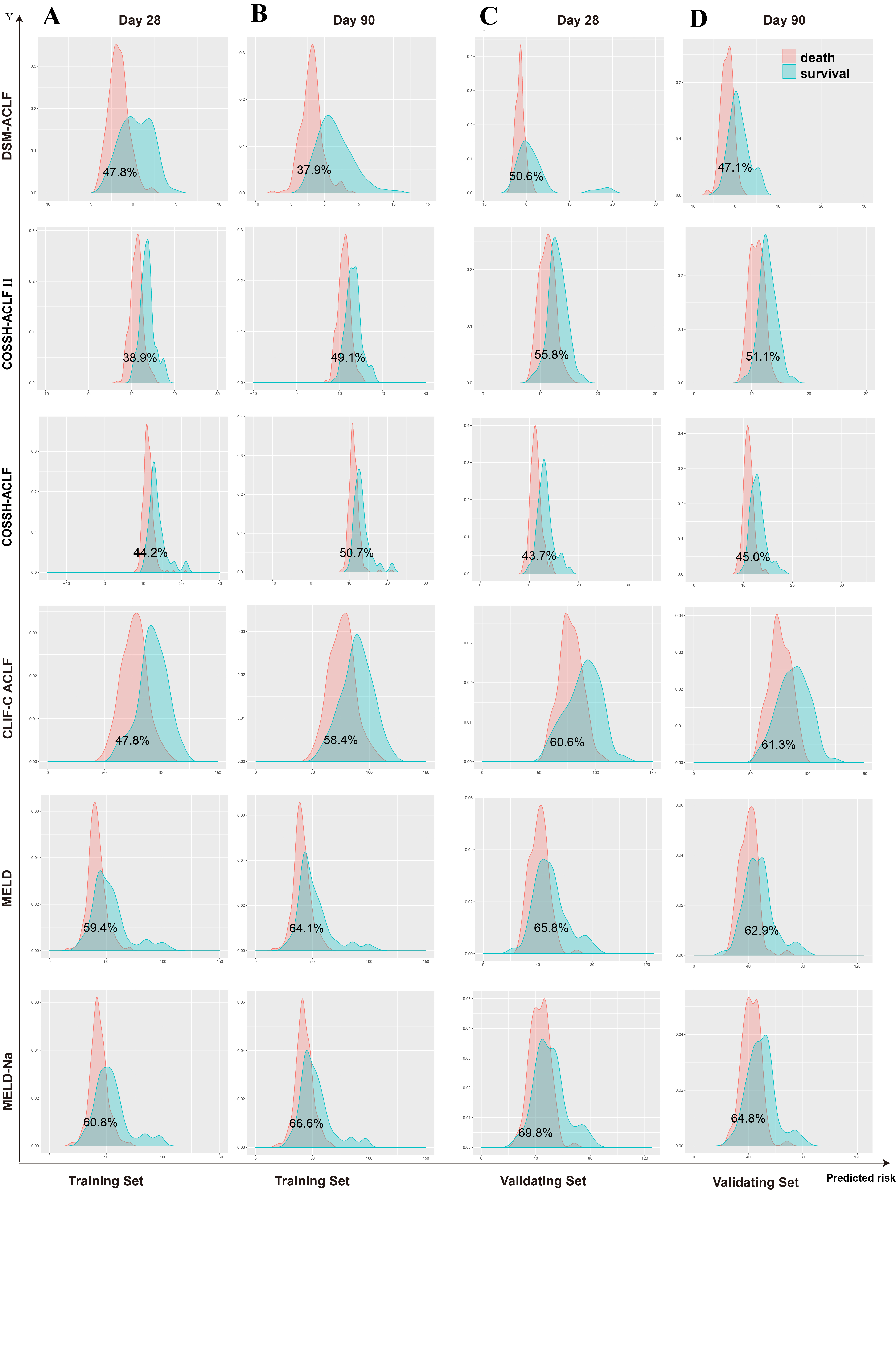

Supplement: Supplementary file 4 — Supplementary Figure 4. [file 41598_2024_63900_MOESM4_ESM.jpg]

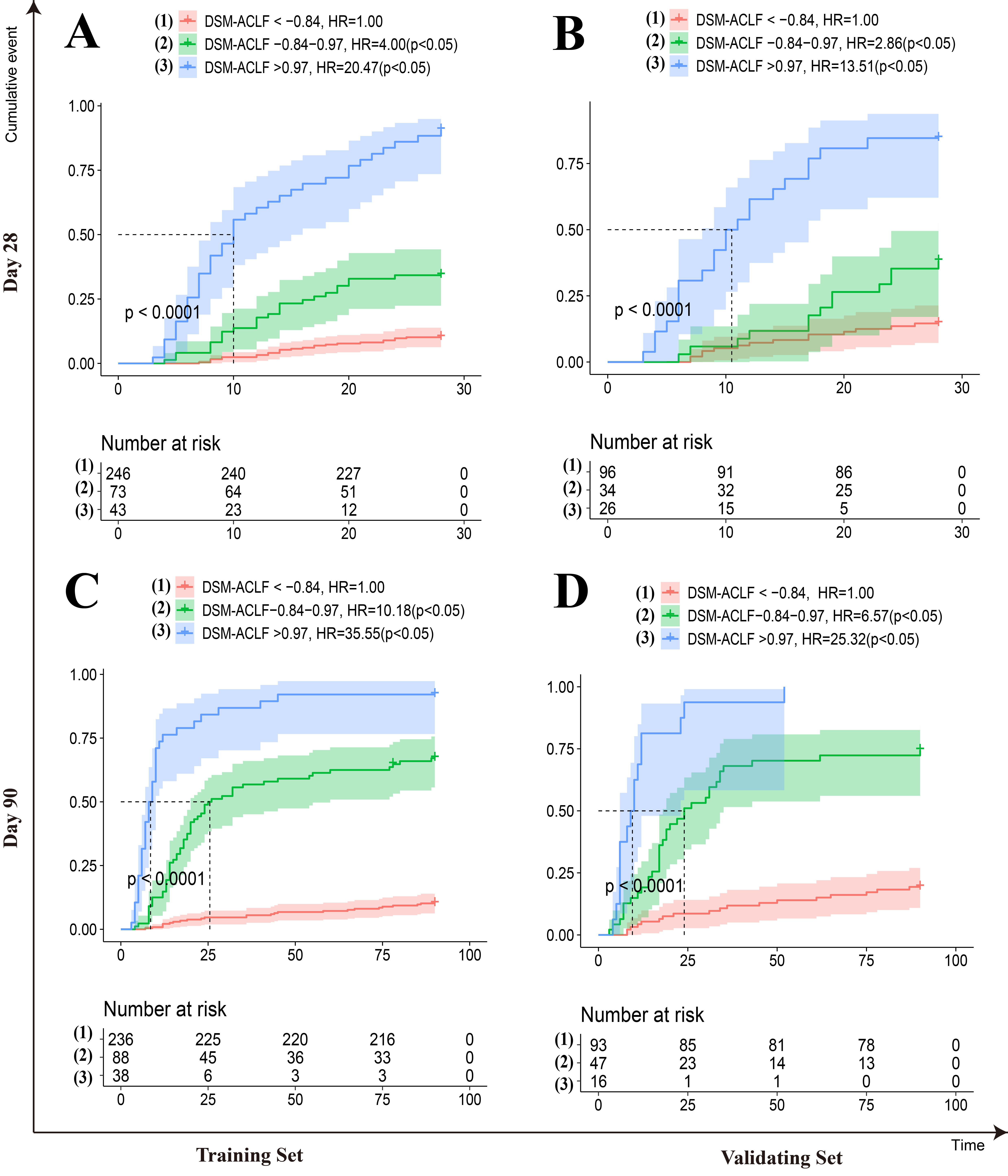

Supplement: Supplementary file 5 — Supplementary Figure 5. [file 41598_2024_63900_MOESM5_ESM.jpg]

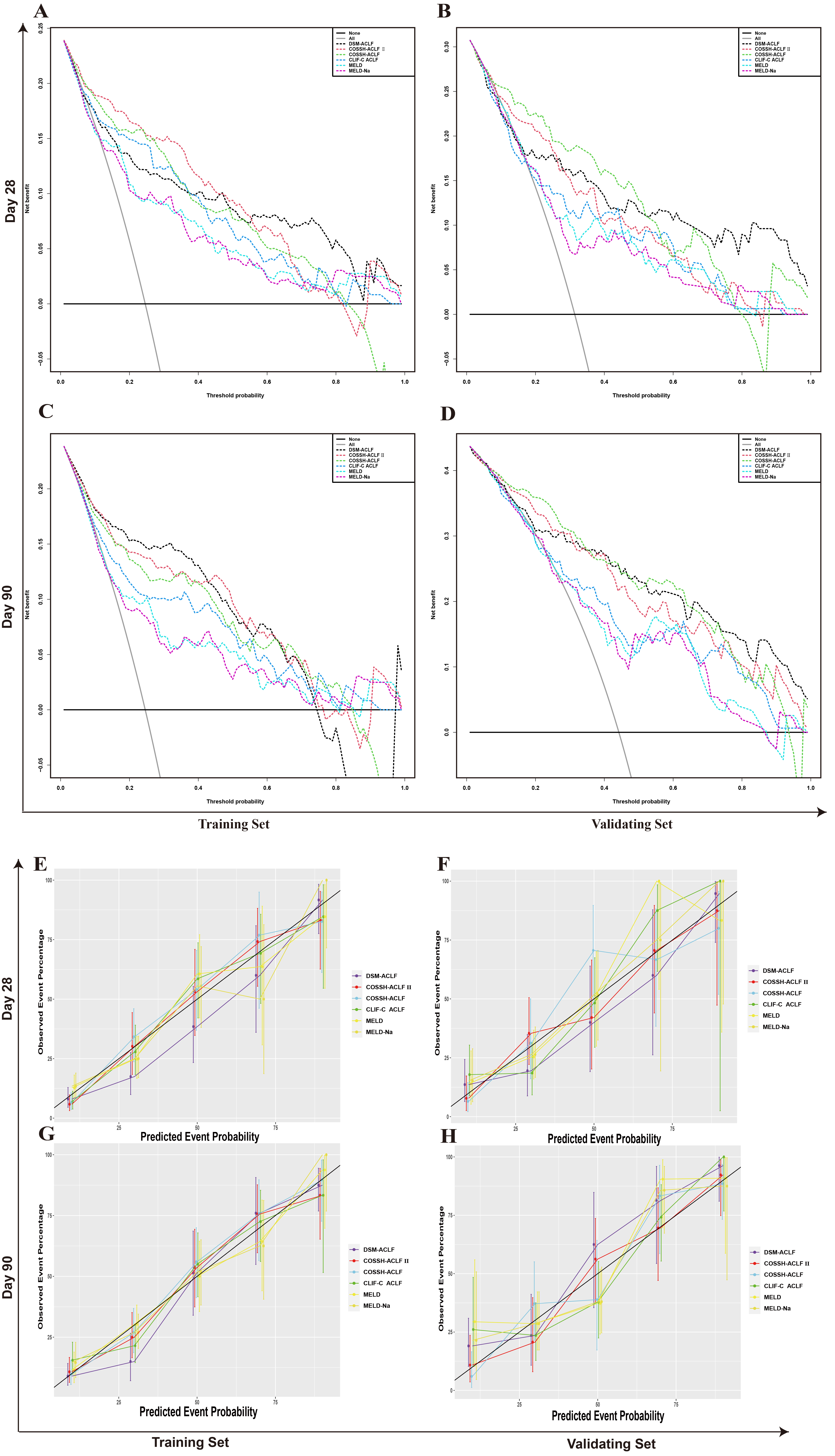

Supplement: Supplementary file 6 — Supplementary Figure 6. [file 41598_2024_63900_MOESM6_ESM.jpg]
