# Supplementary material for: Construction of a novel prognostic scoring model for HBV-ACLF liver failure based on dynamic data
Source: Sci Rep. 2024 Jul 2;14:15198. doi: 10.1038/s41598-024-63900-4 (PMC11219721; doi:10.1038/s41598-024-63900-4)
Supplement: Supplementary file 7 — Supplementary Legends. [file 41598_2024_63900_MOESM7_ESM.docx]

**Supplementary figure 1**: The decision curve (A-D) and calibration curve (E-H) of the new score and the five other scores in day-1.

A、B、E and F: decision and calibration curves of 28 day since admission;

A、C、E and G: in the training set;

C、D、G and H: decision and calibration curves of 90 day since admission;

B、D、F and H: in the validation set.

**Supplementary figure 2**: The decision curve (A-D) and calibration curve (E-H) of the new score and the five other scores in day-3.

A、B、E and F: decision and calibration curves of 28 day since admission;

A、C、E and G: in the training set;

C、D、G and H: decision and calibration curves of 90 day since admission;

B、D、F and H: in the validation set.

**Supplementary figure 3**: Time-dependent ROC curves of the new score and the five other scores in day-(1+3).

A、B: time-dependent ROC curves of 28 day since admission;

A、C：in the training set;

C、D: time-dependent ROC curves of 90 day since admission;

B、D: in the validation set.

**Supplementary figure 4**: Probability density function (PDF) of the new score and the five other scores in day-(1+3).

A、B: in the training set; time-dependent ROC curves of 28 day since admission;

A、C: PDF of 28 day since admission;

C、D: in the validation set;

B、D: PDF of 90 day since admission.

**Supplementary figure 5**: Risk stratification of the new score in day-(1+3).

A、B: Risk stratification of the new score in 28-day since admission;

A、C：in the training set;

C、D: Risk stratification of the new score in 90-day since admission;

B、D: in the validation set.

**Supplementary figure 6**: The decision curve (A-D) and calibration curve (E-H) of the new score and the five other scores in day-(1+3).

A、B、E and F: decision and calibration curves of 28 day since admission;

A、C、E and G: in the training set;

C、D、G and H: decision and calibration curves of 90 day since admission;

B、D、F and H: in the validation set.
