# Supplementary material for: Construction of a novel prognostic scoring model for HBV-ACLF liver failure based on dynamic data
Source: Sci Rep. 2024 Jul 2;14:15198. doi: 10.1038/s41598-024-63900-4 (PMC11219721; doi:10.1038/s41598-024-63900-4)
Supplement: Supplementary file 8 — Supplementary Table 1. [file 41598_2024_63900_MOESM8_ESM.docx]

**Supplementary Table 1. Clinical characteristics of the training and validation groups.**

| **Characteristics** | **Training group(n=362)** | **Validation group(n=156)** | ***P*** |
| --- | --- | --- | --- |
| Male | 311(85.91%) | 129(82.69%) | 0.347 |
| Age (years) | 48.72±11.85 | 49.49±11.75 | 0.587 |
| **Therapies** |  |  |  |
| Vasopressor | 5(1.38%) | 4(2.56%) | 0.345 |
| Mechanical ventilation | 1(0.28%) | 1(0.64%) | 0.539 |
| **Complications** |  |  |  |
| Hepatic encephalopathy | 45(12.43%) | 30(19.23%) | 0.044 |
| Hepatorenal syndrome | 18(4.97%) | 9(5.77%) | 0.708 |
| Infection | 160(44.20%) | 70(44.87%) | 0.888 |
| Gastrointestinal haemorrhage | 22(6.08%) | 11(7.05%) | 0.677 |
| **Severity scores** |  |  |  |
| COSH-ACLF-IIs | 11.64(10.57,12.85) | 11.72(10.47,12.73) | 0.956 |
| COSSH-ACLFs | 11.59(10.73,12.60) | 11.64(10.85,12.74) | 0.480 |
| CLIF-C ACLFs | 79.48(70.93,88.39) | 80.70(72.18,90.09) | 0.448 |
| MELD | 42.20(37.73,48.33) | 42.48(38.04,47.92) | 0.895 |
| MELD-Na | 44.24(39.54,50.51) | 44.66(39.12,50.39) | 0.882 |
| **Vital signs** |  |  |  |
| MAP(mmHg) | 172.25±17.85 | 174.49±19.68 | 0.225 |
| SPO2(%) | 196.00(195.00,197.00) | 196.00(194.00,197.00) | 0.408 |
| **Laboratory data** |  |  |  |
| Total protein(g/L) | 114.80(106.95,121.53) | 116.10(107.70,124.18) | 0.235 |
| Albumin(g/L) | 62.75(58.20,66.43) | 62.65(58.45,67.08) | 0.719 |
| Globulin(g/L) | 50.45(44.00,59.73) | 52.25(45.25,60.85) | 0.256 |
| Alanine aminotransferase(U/L) | 369.00(171.75,804.00) | 408.50(181.75,840.75) | 0.236 |
| Aspartate aminotransferase(U/L) | 263.00(167.50,468.75) | 296.00(172.50,521.50) | 0.249 |
| Alkaline phosphatase(U/L) | 271.00(216.00,319.50) | 262.00(210.25,310.50) | 0.225 |
| Total bile acid(μmol/L) | 486.55(367.13,642.58) | 479.15(352.05,576.45) | 0.248 |
| Total bilirubin(μmol/L) | 609.15(484.23,771.43) | 628.20(494.18,766.38) | 0.896 |
| Direct bilirubin(μmol/L) | 459.75(374.95,603.30) | 481.35(377.95,594.50) | 0.755 |
| Indirect bilirubin(μmol/L) | 134.90(98.03,186.15) | 132.25(95.28,203.95) | 0.850 |
| Glutamyl transferase(U/L) | 148.00(108.00,216.25) | 165.00(111.00,250.75) | 0.073 |
| Creatinine(μmol/L) | 128.00(111.00,151.00) | 129.50(110.25,148.00) | 0.885 |
| Serum urea(mmol/L) | 8.96(7.01,11.71) | 9.03(6.51,11.72) | 0.457 |
| Triglyceride(mmol/L) | 2.47(1.98,3.46) | 2.61(2.00,3.31) | 0.975 |
| Total cholesterol(mmol/L) | 4.74(3.68,5.89) | 4.66(3.78,5.58) | 0.870 |
| High density lipoprotein(mmol/L) | 0.43(0.30,0.60) | 0.44(0.31,0.63) | 0.572 |
| Low density lipoprotein(mmol/L) | 1.77(0.97,2.83) | 2.05(1.09,2.89) | 0.179 |
| Very low density lipoprotein(mmol/L) | 2.00(1.33,3.17) | 2.02(1.24,2.86) | 0.545 |
| Glucose(mmol/L) | 9.35(8.07,11.44) | 9.12(7.82,12.42) | 0.700 |
| K(mmol/L) | 8.13(7.58,8.77) | 8.11(7.63,8.72) | 0.758 |
| Na(mmol/L) | 276.00(273.00,279.00) | 275.50(271.25,280.75) | 0.982 |
| Cl(mmol/L) | 204.00(199.00,208.00) | 204.00(199.25,208.00) | 0.725 |
| Ca(mmol/L) | 4.14(3.99,4.29) | 4.15(4.02,4.29) | 0.738 |
| P(mmol/L) | 1.90(1.62,2.17) | 1.93(1.60,2.16) | 0.464 |
| White blood cell(10^9^/L) | 14.90(11.00,18.75) | 15.20(11.20,18.90) | 0.976 |
| Neutrophil (10^9^/L) | 11.00(7.58,14.80) | 10.45(7.25,14.28) | 0.646 |
| Lymphocyte(10^9^/L) | 2.13(1.56,2.85) | 2.16(1.62,3.03) | 0.238 |
| Monocyte(10^9^/L) | 1.33(0.94,1.84) | 1.41(1.03,1.83) | 0.239 |
| Eosinophil(10^9^/L) | 0.10(0.05,0.16) | 0.10(0.04,0.16) | 0.722 |
| Basophil(10^9^/L) | 0.04(0.03,0.06) | 0.05(0.03,0.07) | 0.165 |
| Red blood cell(10^12^/L) | 7.53(6.66,8.46) | 7.45(6.58,8.55) | 0.893 |
| Haemoglobin (g/L) | 239.00(214.75,262.50) | 241.00(214.25,264.75) | 0.697 |
| Haematocrit (%) | 68.00(60.80,74.83) | 68.60(61.03,74.88) | 0.795 |
| Platelet count(10^9^/L) | 184.00(132.00,260.25) | 193.50(134.25,268.00) | 0.609 |
| INR | 3.72(3.20,4.48) | 3.75(3.23,4.56) | 0.741 |
| Fibrin(g/L) | 2.75(2.23,3.46) | 2.75(2.18,3.34) | 0.713 |
| Prothrombin time(s) | 42.15(36.30,50.13) | 42.80(37.50,50.90) | 0.514 |
| D-Dimer(ug/L) | 4591.00(2222.25,8105.25) | 5706.00(2567.00,9790.25) | 0.121 |
| **LT-free** **mortality** |  |  |  |
| 28-day | 89(24.59%) | 49(31.41%) | 0.107 |
| 90-day | 118(32.60%) | 69(44.23%) | 0.011 |

Note: Data are presented as the means ± SD, medians with (p25, p75), or numbers of patients (percentages). ACLF: acute-on-chronic liver failure; MAP: Mean artery pressure; COSSH-ACLFs: COSSH-ACLF score; COSH-ACLF-IIs: COSSH-ACLF II score; CLIF-C ACLFs: CLIF Consortium ACLF score; MELD: Model for end-stage liver disease. LT: liver transplantation.
