# Supplementary material for: Construction of a novel prognostic scoring model for HBV-ACLF liver failure based on dynamic data
Source: Sci Rep. 2024 Jul 2;14:15198. doi: 10.1038/s41598-024-63900-4 (PMC11219721; doi:10.1038/s41598-024-63900-4)
Supplement: Supplementary file 9 — Supplementary Table 2. [file 41598_2024_63900_MOESM9_ESM.docx]

**Supplementary table 2. Comparison of variable characteristics between the survival group and death groups on day-(1+3).**

| **Characteristics** | **28-day survival group(n=380)** | **28-day death group(n=138)** | ***P*** | | **90-day survival group(n=331)** | | **90-day death**  **group(n=187)** | | ***P*** |
| --- | --- | --- | --- | --- | --- | --- | --- | --- | --- |
| Male | 331(87.11%) | 109(78.99%) | 0.022 | | 292(88.22%) | | 148(79.14%) | | 0.006 |
| Age (years) | 46.99±11.54 | 54.38±10.85 | <0.001 | | 46.38±11.67 | | 53.51±10.65 | | <0.001 |
| **Therapies** |  |  |  | |  | |  | |  |
| Vasopressors | 0 | 9(6.52%) | <0.001 | | 0 | | 9(4.81%) | | <0.001 |
| Mechanical ventilation | 0 | 2(1.45%) | 0.019 | | 0 | | 2(1.07%) | | 0.059 |
| **Complications** |  |  |  | |  | |  | |  |
| Hepatic encephalopathy | 18(4.74%) | 57(41.30%) | <0.001 | | 14(4.23%) | | 61(32.62%) | | <0.001 |
| Hepatorenal syndrome | 8(2.11%) | 19(13.77%) | <0.001 | | 5(1.51%) | | 22(11.76%) | | <0.001 |
| Infection | 157(41.32%) | 73(52.90%) | 0.019 | | 130(39.27%) | | 100(53.48%) | | 0.002 |
| Gastrointestinal haemorrhage | 16(4.21%) | 17(12.32%) | <0.001 | | 10(3.02%) | | 23(12.30%) | | <0.001 |
| **Severity scores** |  |  |  | |  | |  | |  |
| COSH-ACLF-IIs | 11.21(10.18,12.10) | 13.37(12.35,14.29) | <0.001 | | 11.03±1.35 | | 13.44±1.66 | | <0.001 |
| COSSH-ACLFs | 11.20(10.54,11.94) | 13.05(12.35,14.27) | <0.001 | | 11.04(10.48,11.81) | | 12.96(12.05,14.09) | | <0.001 |
| CLIF-C ACLFs | 76.66±10.9 | 91.3±13.61 | <0.001 | | 75.81(68.43,83.62) | | 12.81(11.86,13.81) | | <0.001 |
| MELD | 40.75(36.73,45.33) | 49.07(42.41,57.79) | <0.001 | | 40.17(36.01,44.66) | | 47.64(41.95,54.20) | | <0.001 |
| MELD-Na | 42.63(38.69,47.78) | 51.75(44.25,58.22) | <0.001 | | 42.11(38.25,47.18) | | 49.43(43.62,55.84) | | <0.001 |
| **Vital signs** |  |  |  | |  | |  | |  |
| MAP(mmHg) | 172.11±17.65 | 175.17±20.31 | 0.118 | | 172.22±17.00 | | 174.18±20.71 | | 0.148 |
| SPO2(%) | 196.00(195.00,197.00) | 195.00(193.00,197.00) | 0.001 | | 196.00(195.00,197.00) | | 195.00(194.00,197.00) | | 0.014 |
| **Laboratory data** |  |  |  | |  | |  | |  |
| Total protein(g/L) | 115.20(107.30,122.18) | 115.30(105.75,122.38) | 0.781 | | 115.40(107.40,122.30) | | 114.70(105.80,122.20) | | 0.468 |
| Albumin(g/L) | 62.90(58.20,66.80) | 62.10(58.63,66.43) | 0.541 | | 63.20(58.50,67.00) | | 61.60(58.10,66.20) | | 0.048 |
| Globulin(g/L) | 50.90(44.73,59.75) | 50.35(42.48,62.03) | 0.792 | | 50.80(44.50,60.20) | | 51.00(43.40,60.70) | | 0.931 |
| Alanine aminotransferase(U/L) | 369.00(176.00,790.50) | 419.00(188.75,849.00) | 0.445 | | 372.00(181.00,797.00) | | 389.00(172.00,843.00) | | 0.957 |
| Aspartate aminotransferase(U/L) | 258.50(163.25,412.00) | 353.50(193.00,596.50) | 0.001 | | 257.00(164.00,412.00) | | 330.00(174.00,596.00) | | 0.004 |
| Alkaline phosphatase(U/L) | 268.00(215.25,313.00) | 275.00(216.00,321.75) | 0.426 | | 269.00(215.00,314.00) | | 268.00(216.00,314.00) | | 0.823 |
| Total bile acid(μmol/L) | 475.05(361.50,603.65) | 501.70(351.93,682.70) | 0.380 | | 475.50(366.20,604.00) | | 497.20(345.70,645.20) | | 0.680 |
| Total bilirubin(μmol/L) | 583.00(475.60,729.45) | 710.80(564.38,890.15) | <0.001 | | 564.10(463.00,707.50) | | 712.30(568.90,880.10) | | <0.001 |
| Direct bilirubin(μmol/L) | 450.00(365.38,566.55) | 549.00(405.98,669.50) | <0.001 | | 440.70(362.60,548.00) | | 550.00(422.00,667.10) | | <0.001 |
| Indirect bilirubin(μmol/L) | 124.95(89.15,168.40) | 167.00(124.95,237.15) | <0.001 | | 119.10(85.40,163.50) | | 163.90(125.00,231.80) | | <0.001 |
| Glutamyl transferase(U/L) | 159.00(117.00,228.75) | 133.50(94.00,216.00) | 0.007 | | 159.00(117.00,224.00) | | 137.00(100.00,228.00) | | 0.022 |
| Creatinine(μmol/L) | 128.50(112.00,145.75) | 132.50(105.50,169.25) | 0.212 | | 128.00(112.00,145.00) | | 132.00(106.00,167.00) | | 0.190 |
| Serum urea(mmol/L) | 8.53(6.68,10.76) | 10.91(8.13,17.00) | <0.001 | | 8.36(6.46,10.36) | | 10.54(8.16,14.99) | | <0.001 |
| Triglyceride(mmol/L) | 2.67(2.12,3.64) | 2.12(1.83,2.80) | <0.001 | | 2.73(2.14,3.64) | | 2.19(1.85,2.90) | | <0.001 |
| Total cholesterol(mmol/L) | 4.95(3.98,5.97) | 4.23(3.15,5.22) | <0.001 | | 4.98(4.03,5.99) | | 4.28(3.16,5.29) | | <0.001 |
| High density lipoprotein(mmol/L) | 0.41(0.29,0.60) | 0.46(0.34,0.65) | 0.017 | | 0.41(0.29,0.60) | | 0.45(0.32,0.63) | | 0.083 |
| Low density lipoprotein(mmol/L) | 1.84(0.96,2.88) | 1.88(1.20,2.68) | 0.820 | | 1.85(1.00,2.88) | | 1.83(1.05,2.78) | | 0.852 |
| Very low density lipoprotein(mmol/L) | 2.23(1.52,3.30) | 1.38(0.93,2.22) | <0.001 | | 2.29(1.52,3.31) | | 1.57(1.02,2.41) | | <0.001 |
| Glucose(mmol/L) | 9.25(8.04,11.23) | 9.86(7.77,14.18) | 0.135 | | 9.26(8.04,11.16) | | 9.34(7.80,13.97) | | 0.190 |
| K(mmol/L) | 8.12(7.63,8.73) | 8.13(7.38,8.85) | 0.833 | | 8.14(7.66,8.76) | | 8.00(7.51,8.77) | | 0.206 |
| Na(mmol/L) | 276.00(273.00,279.00) | 275.50(270.75,280.00) | 0.842 | | 276.00(273.00,279.00) | | 275.00(271.00,280.00) | | 0.543 |
| Cl(mmol/L) | 204.00(200.00,208.00) | 202.00(196.00,208.00) | 0.003 | | 205.00(201.00,208.00) | | 202.00(197.00,208.00) | | <0.001 |
| Ca(mmol/L) | 4.15(4.01,4.29) | 4.14(3.97,4.31) | 0.964 | | 4.15(4.00,4.28) | | 4.15(3.99,4.30) | | 0.820 |
| P(mmol/L) | 1.92(1.65,2.16) | 1.80(1.52,2.17) | 0.078 | | 1.94(1.66,2.17) | | 1.79(1.52,2.13) | | 0.004 |
| White blood cell(10^9^/L) | 14.35(10.63,18.18) | 16.93(12.65,22.23) | <0.001 | | 14.40(10.50,18.20) | | 16.10(12.00,21.00) | | <0.001 |
| Neutrophil (10^9^/L) | 10.10(7.00,13.78) | 12.30(8.98,17.53) | <0.001 | | 10.00(6.90,13.60) | | 11.80(8.50,16.50) | | <0.001 |
| Lymphocyte(10^9^/L) | 2.25(1.61,2.94) | 1.92(1.34,2.55) | 0.002 | | 2.29(1.65,2.99) | | 1.92(1.39,2.60) | | <0.001 |
| Monocyte(10^9^/L) | 1.28(0.94,1.75) | 1.54(1.09,2.20) | <0.001 | | 1.28(0.95,1.76) | | 1.47(1.02,2.10) | | 0.004 |
| Eosinophil(10^9^/L) | 0.11(0.06,0.17) | 0.07(0.04,0.15) | | 0.004 | | 0.11(0.06,0.17) | | 0.07(0.04,0.16) | 0.003 |
| Basophil(10^9^/L) | 0.04(0.03,0.07) | 0.04(0.02,0.06) | | 0.181 | | 0.05(0.03,0.07) | | 0.04(0.02,0.06) | 0.018 |
| Red blood cell(10^12^/L) | 7.62(6.76,8.56) | 7.19(6.44,8.16) | | 0.005 | | 7.64(6.80,8.57) | | 7.28(6.47,8.26) | 0.012 |
| Haemoglobin (g/L) | 242.00(216.00,264.00) | 228.50(205.25,261.00) | | 0.014 | | 242.00(219.00,264.00) | | 232.00(208.00,261.00) | 0.015 |
| Haematocrit (%) | 68.75(61.53,75.18) | 66.05(58.95,73.63) | | 0.029 | | 68.90(62.20,75.00) | | 66.10(58.60,74.00) | 0.017 |
| Platelet count(10^9^/L) | 191.50(136.25,269.00) | 178.50(118.00,242.75) | | 0.007 | | 199.00(143.00,274.00) | | 174.00(119.00,233.00) | <0.001 |
| INR | 3.53(3.11,4.04) | 4.65(3.94,5.95) | | <0.001 | | 3.45(3.08,3.96) | | 4.45(3.81,5.32) | <0.001 |
| Fibrin(g/L) | 2.94(2.39,3.54) | 2.33(1.79,2.83) | | <0.001 | | 2.95(2.39,3.55) | | 2.46(1.90,3.09) | <0.001 |
| Prothrombin time(s) | 40.25(35.50,45.08) | 52.40(44.48,66.28) | | <0.001 | | 39.50(35.00,44.70) | | 49.40(43.00,59.80) | <0.001 |
| D-Dimer(ug/L) | 3874.50(1790.00,6597.75) | 7987.50(5614.50,12309.75) | | <0.001 | | 3790.00(1709.00,6447.00) | | 7604.00(4244.00,12093.00) | <0.001 |

Note: Data are presented as the means ± SD, medians with (p25, p75), or numbers of patients (percentages).

ACLF: acute-on-chronic liver failure; MAP: Mean artery pressure; COSSH-ACLFs: COSSH-ACLF score; COSH-ACLF-IIs: COSSH-ACLF II score; CLIF-C ACLFs: CLIF Consortium ACLF score; MELD: Model for end-stage liver disease. LT: liver transplantation.
